# *Pseudomonas aeruginosa* MifS-MifR Two-Component System Is Specific for α-Ketoglutarate Utilization

**DOI:** 10.1371/journal.pone.0129629

**Published:** 2015-06-26

**Authors:** Gorakh Tatke, Hansi Kumari, Eugenia Silva-Herzog, Lourdes Ramirez, Kalai Mathee

**Affiliations:** 1 Department of Biological Sciences, College of Arts & Sciences, Florida International University, Miami, Florida, United States of America; 2 Department of Molecular Microbiology and Infectious Diseases, Herbert Wertheim College of Medicine, Florida International University, Miami, Florida, United States of America; University of North Dakota, UNITED STATES

## Abstract

*Pseudomonas aeruginosa* is a Gram-negative, metabolically versatile opportunistic pathogen that elaborates a multitude of virulence factors, and is extraordinarily resistant to a gamut of clinically significant antibiotics. This ability, in part, is mediated by two-component regulatory systems (TCS) that play a crucial role in modulating virulence mechanisms and metabolism. MifS (PA5512) and MifR (PA5511) form one such TCS implicated in biofilm formation. MifS is a sensor kinase whereas MifR belongs to the NtrC superfamily of transcriptional regulators that interact with RpoN (σ^54^). In this study we demonstrate that the *mifS* and *mifR* genes form a two-gene operon. The close proximity of *mifSR* operon to *poxB* (*PA5514*) encoding a ß-lactamase hinted at the role of MifSR TCS in regulating antibiotic resistance. To better understand this TCS, clean in-frame deletions were made in *P*. *aeruginosa* PAO1 creating PAO∆*mifS*, PAO∆*mifR* and PAO∆*mifSR*. The loss of *mifSR* had no effect on the antibiotic resistance profile. Phenotypic microarray (BioLOG) analyses of PAO∆*mifS* and PAO∆*mifR* revealed that these mutants were unable to utilize C_5_-dicarboxylate α-ketoglutarate (α-KG), a key tricarboxylic acid cycle intermediate. This finding was confirmed using growth analyses, and the defect can be rescued by *mifR* or *mifSR* expressed in *trans*. These *mifSR* mutants were able to utilize all the other TCA cycle intermediates (citrate, succinate, fumarate, oxaloacetate or malate) and sugars (glucose or sucrose) except α-KG as the sole carbon source. We confirmed that the *mifSR* mutants have functional dehydrogenase complex suggesting a possible defect in α-KG transport. The inability of the mutants to utilize α-KG was rescued by expressing *PA5530*, encoding C_5_-dicarboxylate transporter, under a regulatable promoter. In addition, we demonstrate that besides MifSR and PA5530, α-KG utilization requires functional RpoN. These data clearly suggests that *P*. *aeruginosa* MifSR TCS is involved in sensing α-KG and regulating its transport and subsequent metabolism.

## Introduction

*Pseudomonas aeruginosa* is a metabolically versatile, Gram-negative opportunistic pathogen that is well known for its extensive spatio-temporal distribution [1]. It is a dominant nosocomial pathogen capable of causing acute and chronic infections in immunocompromised and immunosuppressed patients [2,3]. In particular, patients with AIDS, severe burn wounds, cystic fibrosis (CF), chronic obstructive pulmonary disease (COPD), non-CF bronchiectasis and neutropenia are predisposed to *P*. *aeruginosa* infections [1,4–7]. *P*. *aeruginosa* chronic pulmonary infections are characterized by intensive bronchial neutrophilic inflammation resulting in respiratory failure [8,9], a major cause of fatality in CF patients [10]. Moreover, *P*. *aeruginosa* is associated with keratitis [11] and chronic suppurative otitis media [12] leading to visual impairment and deafness [13,14]. *P*. *aeruginosa* possess numerous virulence factors, both cell-surface associated and secretory, which significantly contribute to its pathogenesis [15]. Effective treatment of *P*. *aeruginosa* infections is impeded by its extraordinary intrinsic and acquired resistance to numerous clinically important antibiotics [16]. Thus, antibiotic resistance and expression of multi-determinant virulence factors are two critical hallmarks in *P*. *aeruginosa* infections that make it an intimidating pathogen.

Successful infection and disease progression depends significantly on the ability of any pathogen to effectively utilize available nutrients that are essential for its growth and survival. *P*. *aeruginosa* is renowned for its extraordinary ability to utilize wide range of organic compounds such as carbohydrates, amino acids, fatty acids, mono- and polyalcohols, di- and tri-carboxylic acids as sources of carbon, nitrogen and energy [1]. However, unlike other bacteria where glucose is the preferred carbon source [17,18], *P*. *aeruginosa* preferentially utilizes tricarboxylic acid (TCA) cycle intermediates [19,20], specifically, C_4_-dicarboxylates of the TCA cycle such as malate, fumarate and succinate [19–21].

The TCA cycle is an amphibolic pathway that serves two main purposes: energy-generation in aerobic organisms (catabolism), and the generation of intermediates to serve as biosynthetic precursors for fatty acid, amino acid and carbohydrate synthesis (anabolism) [22]. The metabolic intermediates of the TCA cycle consist of a group of organic anions that include C_4_-dicarboxylates (succinate, fumarate, malate and oxaloacetate), C_5_-dicarboxylates (alpha-ketoglutarate (α-KG)) and C_6_-tricarboxylates (citrate, isocitrate) [23,24]. However, the role of TCA cycle intermediates is not restricted to energy metabolism or to serve as biosynthetic precursors. In the recent years, TCA cycle intermediates, in-particular, succinate and/or α-KG have gained significant importance as biological signaling molecules in variety of organisms including, bacteria [25], animals [26] and plants [27].

Sensing the available nutrients is a prerequisite for mobilizing the uptake systems. Bacterial two-component systems (TCSs), involving a membrane-bound histidine sensor kinase (HK) and a cytoplasmic response regulator (RR) play an integral part in bacteria’s ability to sense physiological cues. In response to stimuli, the sensor autophosporylates at a conserved histidine residue at the C-terminus, and subsequently the phosphate is transferred to an aspartate residue at the N-terminus of the RR [28–30]. TCSs in *Bacillus subtilis*, *Corynebacterium glutamicum*, *Escherichia coli*, *Klebsiella pneumoniae*, *Rhizobium meliloti* and *Rhizobium leguminosarum* have been shown to regulate extracellular C_4_-dicarboxylates and tricarboxylates transport [28,31–36]. Of these, DctB-DctD in *R*. *meliloti* is an extensively studied TCS, which in coordination with sigma factor RpoN(σ^54^) regulates the extracellular transport of C_4_-dicarboxylates succinate, fumarate and malate [37,38].

Three TCS protein pairs in *P*. *aeruginosa* namely, PA5165/PA5166 (DctB/DctD), PA5512/PA5511 (MifS/MifR) and PA1336/PA1335 have been identified to be homologous to the *Rhizobium* C_4_-dicarboxylate transport regulatory DctB/DctD [39]. Amongst the three, very little is known of PA1336/PA1335. The PA5165/PA5166 (DctB/DctD) TCS has been demonstrated to regulate the transport of C_4_-dicarboxylates, succinate, fumarate and malate in coordination with the sigma factor RpoN (σ^54^) [39]. The SK MifS (65.3 kDa) and RR MifR (49.6 kDa) share 51% and 69% sequence identity to the *R*. *meliloti* DctB and DctD, respectively [40]. The RR MifR is involved in regulating the maturation stage of *P*. *aeruginosa* biofilm formation as *mifR* deficient mutants fail to form microcolonies [41]. Later studies reported the interdependence of pyruvate fermentation and functional MifR in supporting microcolony formation [42]. However, the mechanism by which MifR is activated in this process remains obscure and no relation with HK MifS has been established. Using clean in-frame deletion mutants of the *mifS*, *mifR* and *mifSR* genes we show that MifSR TCS regulates *P*. *aeruginosa* α-KG transport and requires functional RpoN.

## Results

### *mifS* and *mifR* are a part of a two-gene operon

**Fig 1:**
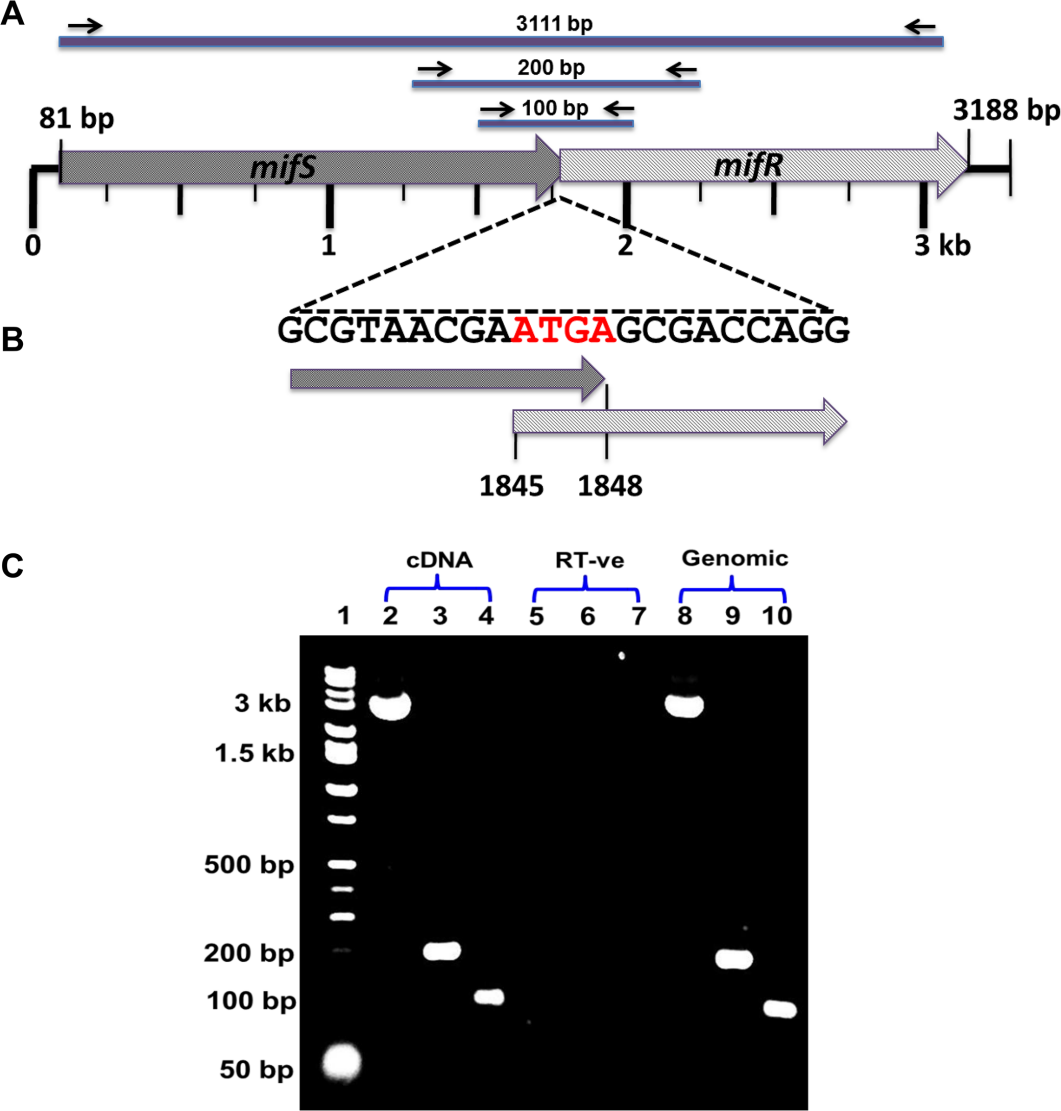
Genome organization of the *mifSR* gene locus. In *P*. *aeruginosa* PAO1 the *mifR* (*PA5511*) ORF has a translation start codon (ATG) overlapping the *mifS* (PA5512) termination codon (TGA), denoted in red (B), suggesting that the *mifS* and *mifR* genes are physically linked. The cDNA amplification of the intergenic region spanning the *mifS* and *mifR* genes using GDT_cotrans F1-R1 and GDT_cotrans F2-R2 primers (Table 1) confirm that the two genes *mifS* and *mifR* are co-transcribed and form an operon (C).

In eubacteria, the genes that encode a HK and its cognate RR are often linked and are co-transcribed [30]. Our sequence analysis of *P*. *aeruginosa* PAO1 genome revealed that *mifS* (*PA5512)* and *mifR* (*PA5511*) are adjacent to each other, in the same orientation. The predicted translation start site of *mifR* ORF overlaps with *mifS* translation termination codon indicating that they are cotranscribed (Fig 1A and 1B). To determine if these two genes form an operon, cDNA across the intergenic regions spanning *mifS* and *mifR* was amplified using GDT_cotransF1-R1 and GDT_cotransF2-R2 primers (see Materials and Methods). As expected, 200 bp and 100 bp products were detected when using primers that span the overlapping region (Fig 1C, Lane 3 and Lane 4). These results confirm that *mifS* and *mifR* are a part of a two-gene operon. As controls, the *mifSR* genes were also amplified (Fig 1C, Lane 2).

### Loss of *mifS* and *mifR* did not affect antibiotic resistance

To identify the role of MifSR TCS, clean in-frame deletion mutants of *mifS*, *mifR and mifSR* were constructed in the prototypic *P*. aeruginosa PAO1. Henceforth they will be referred to as PAO∆*mifS*, PAO∆*mifR* and PAO∆*mifSR*, respectively. For complementation studies, recombinant plasmids containing the entire *mifR*, *mifS* and *mifSR* genes were constructed. The complementing plasmids with the genes are called pMifS, pMifR and pMifSR. These plasmids were introduced into the respective mutant strains.

**Fig 2:**
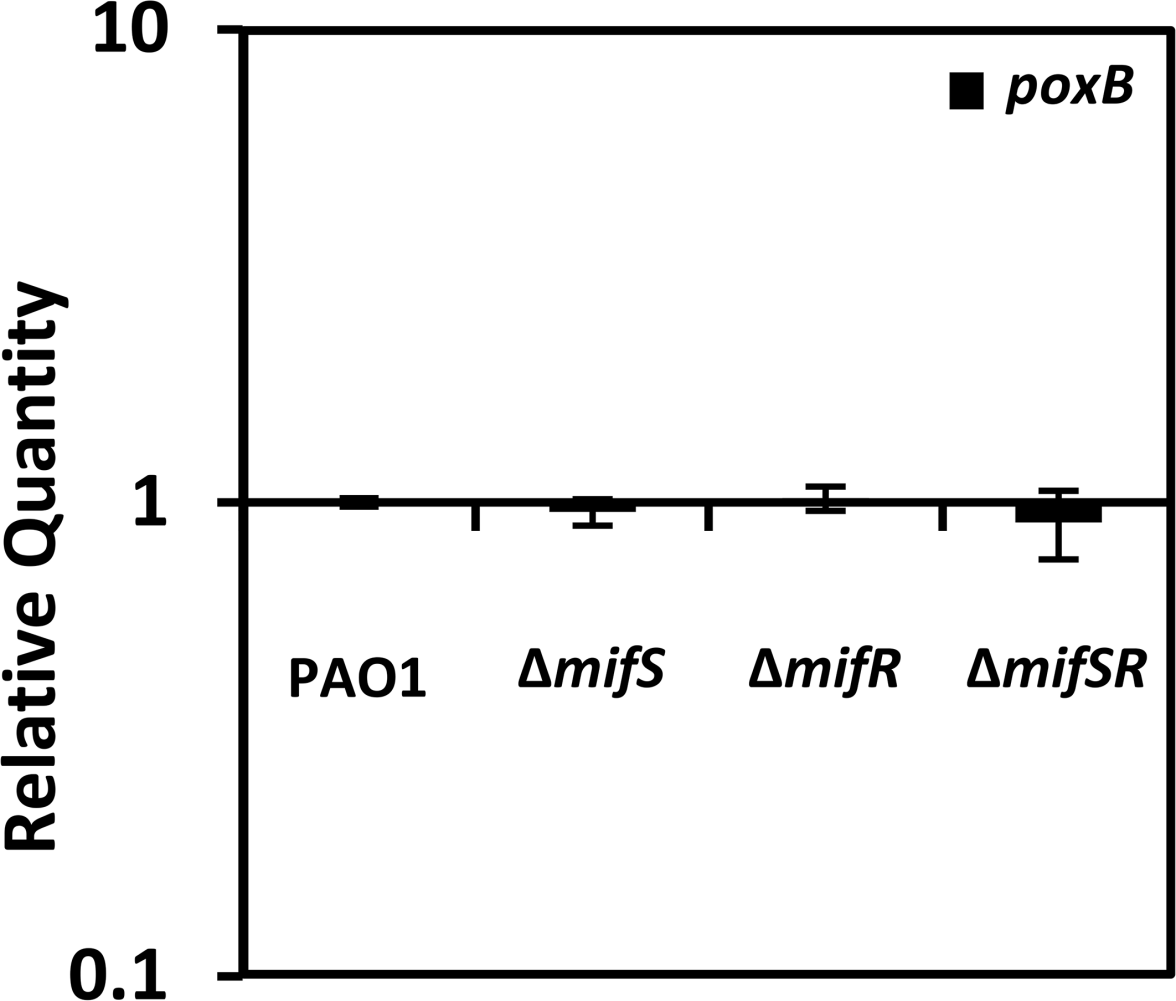
Expression of *poxB* (*PA5514*) in *mifSR* mutants. Expression of *poxB* (*PA5514*) was tested in *mifSR* mutants relative to PAO1. Data was normalized to expression in PAO1. Bars above or below the line represents up- and down-regulation, respectively and the bars indicate standard errors. The *clpX* gene (*PA1802*) was used as the housekeeping control. There was no statistically significant difference (*p*-value > 0.05) between the wild type PAO1 and *mifSR* mutant strains as determined by one-way ANOVA and student’s unpaired *t* test.

Previous studies in our lab postulated that the MifSR TCS system, found 81-bp upstream of the *pox* operon, may contribute to *P*. *aeruginosa* ß-lactam resistance [43] as the genes regulated by TCS tend to be co-located on the chromosome [30]. However, MIC analyses using E-test and micro-dilution methods showed that the loss of these genes did not affect the antibiotic resistance profile when compared to the parent strain, *P*. *aeruginosa* PAO1(Data not shown). Further, qRT-PCR studies showed that deletion of *mifS*, *mifR* and *mifSR* had no effect on the expression of *poxB* compared to the parent PAO1 (Fig 2).

### *mifS*, *mifR* and *mifSR* mutants failed to grow in the presence of α-KG

**Fig 3:**
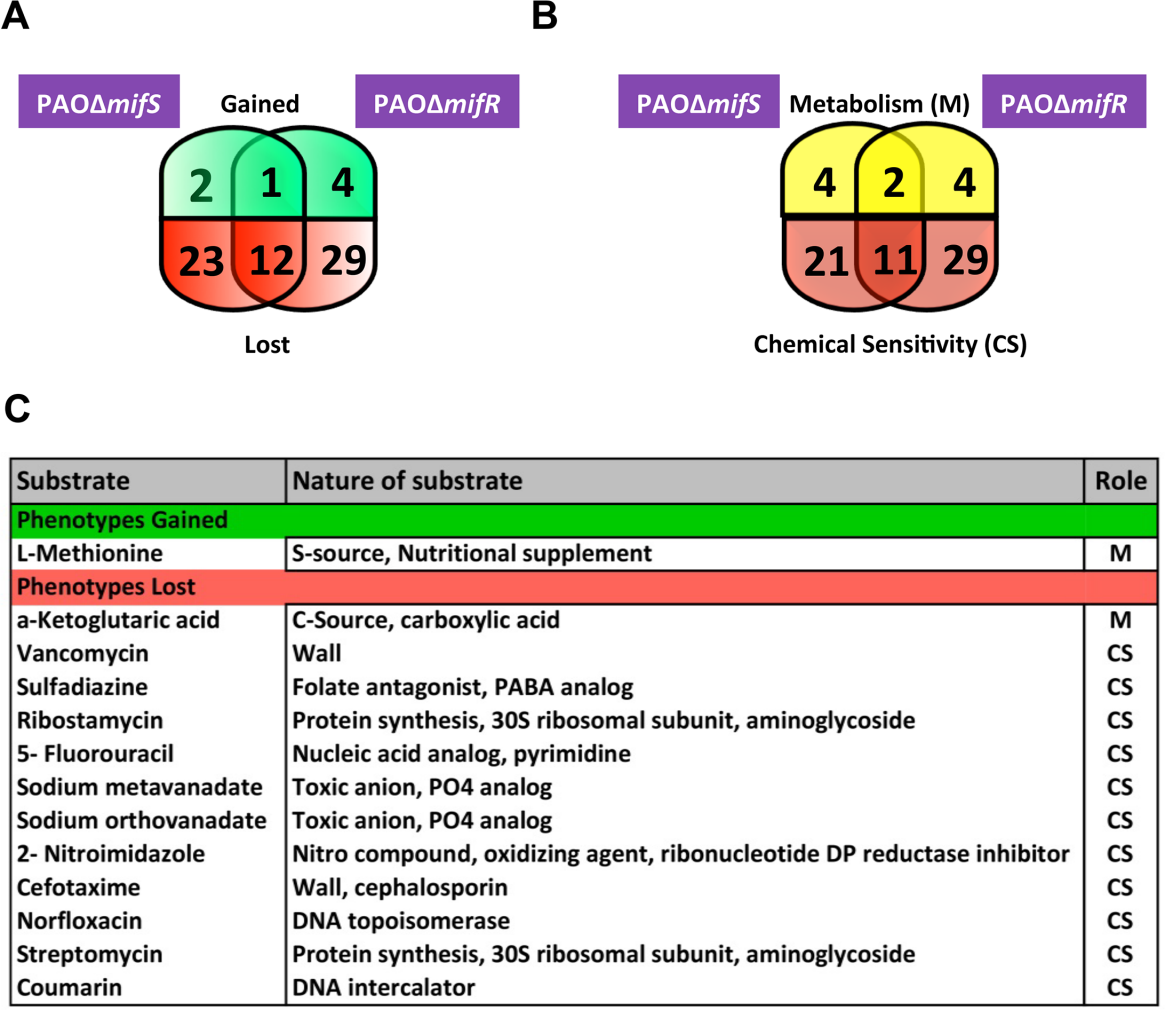
*mifS* and *mifR* dependent phenotypes. To identify the role of *P*. *aeruginosa mifSR* TCS, comparative phenotypic microarray of PAO∆*mifS*, PAO∆*mifR* mutants and wild-type PAO1 strain was performed at BioLOG Inc. (Hayward, CA, USA). Venn diagram of differentially regulated phenotypes of the mutants compared to their isogenic parent PAO1, showing gain of function or loss of function phenotypes (A). Phenotypic differences were further classified based on metabolic and chemical sensitivity properties (B). The phenotypes common to both *mifS* and *mifR* mutants are listed (C).

**Fig 4:**
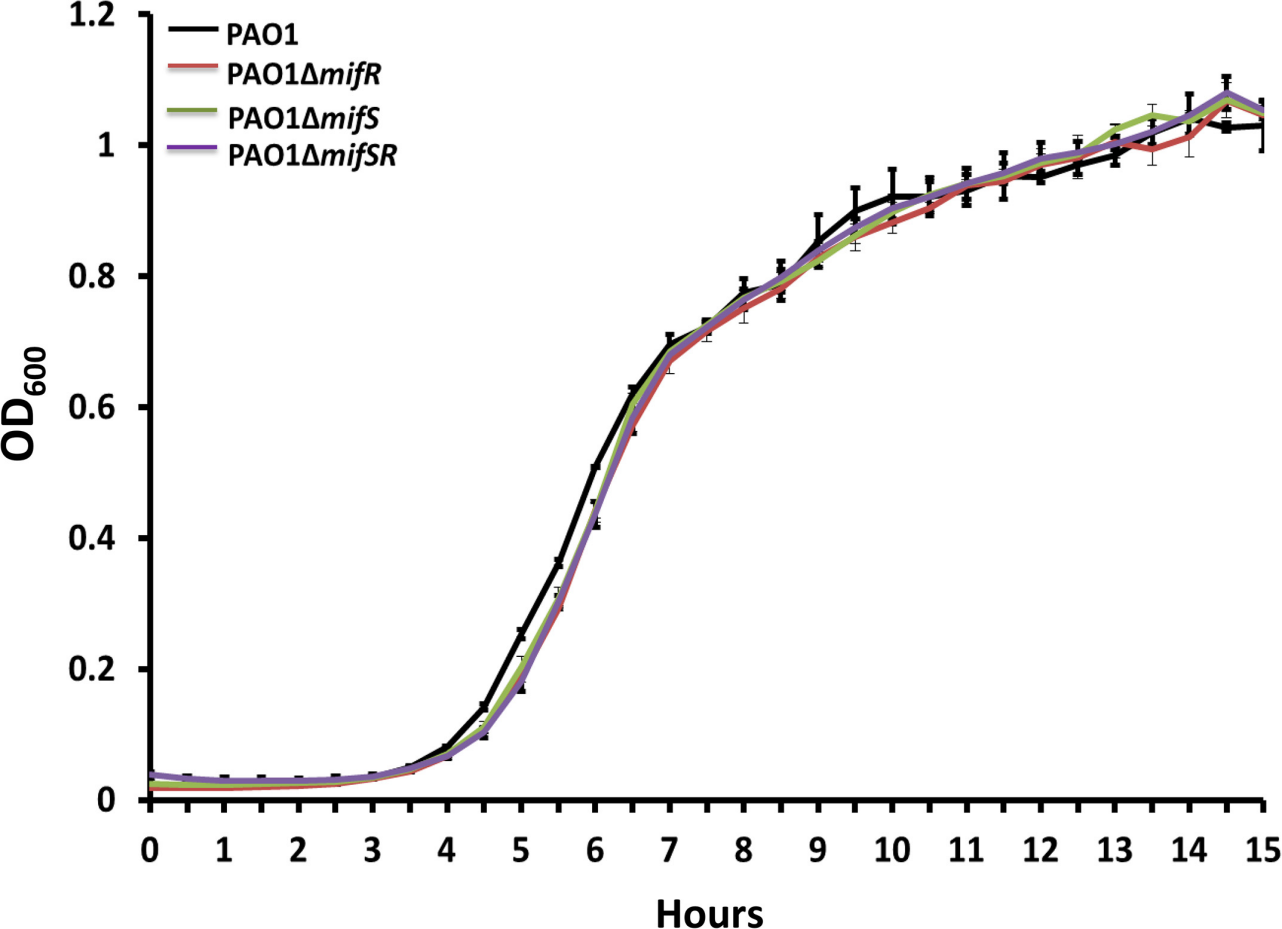
Growth curve analysis in the presence to methionine. Growth curves of *P*. *aeruginosa* wild-type PAO1 and *mifSR* mutants in M9 minimal media supplemented with glucose (30 mM) and methionine (5 mM) as carbon and nitrogen source.

The PAO∆*mifS*, PAO∆*mifR* and PAO∆*mifSR* mutants exhibited no discernible phenotype compared to the parent PAO1 when tested for growth, swimming, swarming, twitching motility (LB media), pyocyanin production (LB & King’s A media), pyoverdine production (LB & King’s B Media), congo red binding assay (CR media) and antibiotic resistance (MH media) (Data not shown). Hence, a comparative phenotypic microarray analysis was performed with the wild-type PAO1, PAO∆*mifR* and PAO∆*mifS* mutants (BioLOG Inc.). Out of approximately 2000 metabolic and chemical sensitivity assays tested, PAO∆*mifR* exhibited four gain-of-function and 29 loss-of-function phenotypes whereas PAO∆*mifS* exhibited two gain-of-function and 23 loss-of-function phenotypes (Fig 3A). A single gain of function phenotype shared between PAO∆*mifS* and PAO∆*mifR*, was the ability to utilize L-methionine. When metabolism and chemical sensitivity were compared, the mutants appear more sensitive to various antibiotics (Fig 3B). However, none of these were reproducible in the lab in the MH media. The loss of *mifS* and *mifR* resulted in differential phenotype in the presence of six metabolites, amongst which, two were common to both *mifS* and *mifR* mutants (Fig 3B). The shared metabolic phenotypes involved the utilization of L-methionine and α-KG (Fig 3C). Compared to the parent PAO1, the mutants did not exhibit any growth increase when provided with L-methionine (Fig 4). This could be simply due to the difference in culture conditions and BioLOG proprietary media.

**Fig 5:**
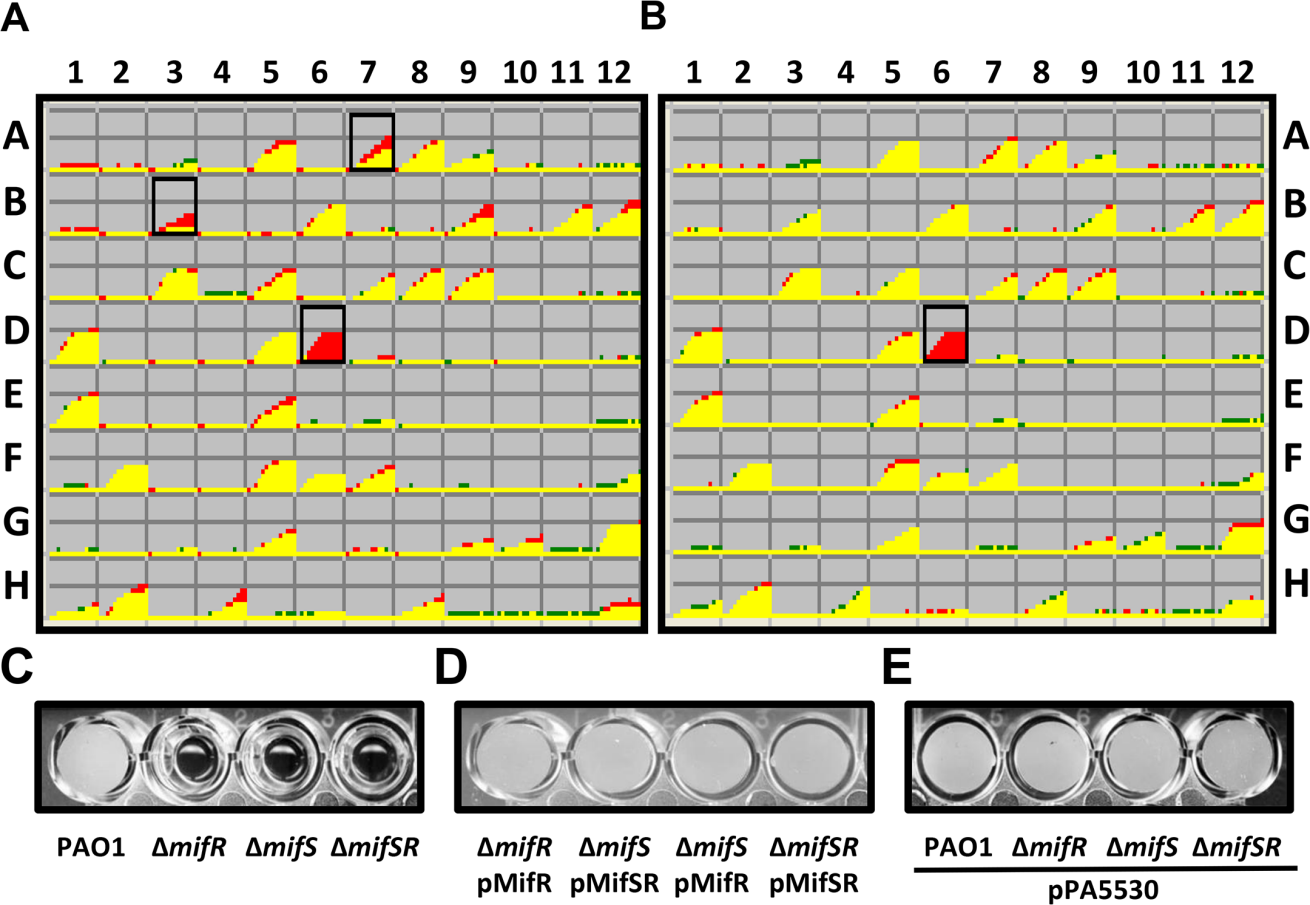
Phenotypic microarrays of PAOΔ*mifS* and PAOΔ*mifR* mutants. The loss of *mifS* and *mifR* results in a growth deficient phenotype in the presence of α-KG as a sole carbon source, as depicted by BioLOG plate PM1, well D6 (A and B). Loss of growth phenotype was confirmed by growing PAO1, PAO∆*mifS*, PAO∆*mifR* and PAO∆*mifSR* mutants in M9 minimal media with α-KG (30 mM) for 18 to 24 h at 37°C (C). The growth defect was rescued by expressing *mifR* and *mifSR* genes (D) and the gene encoding the α-KG specific transporter PA5530 (E) in *trans*.

**Fig 6:**
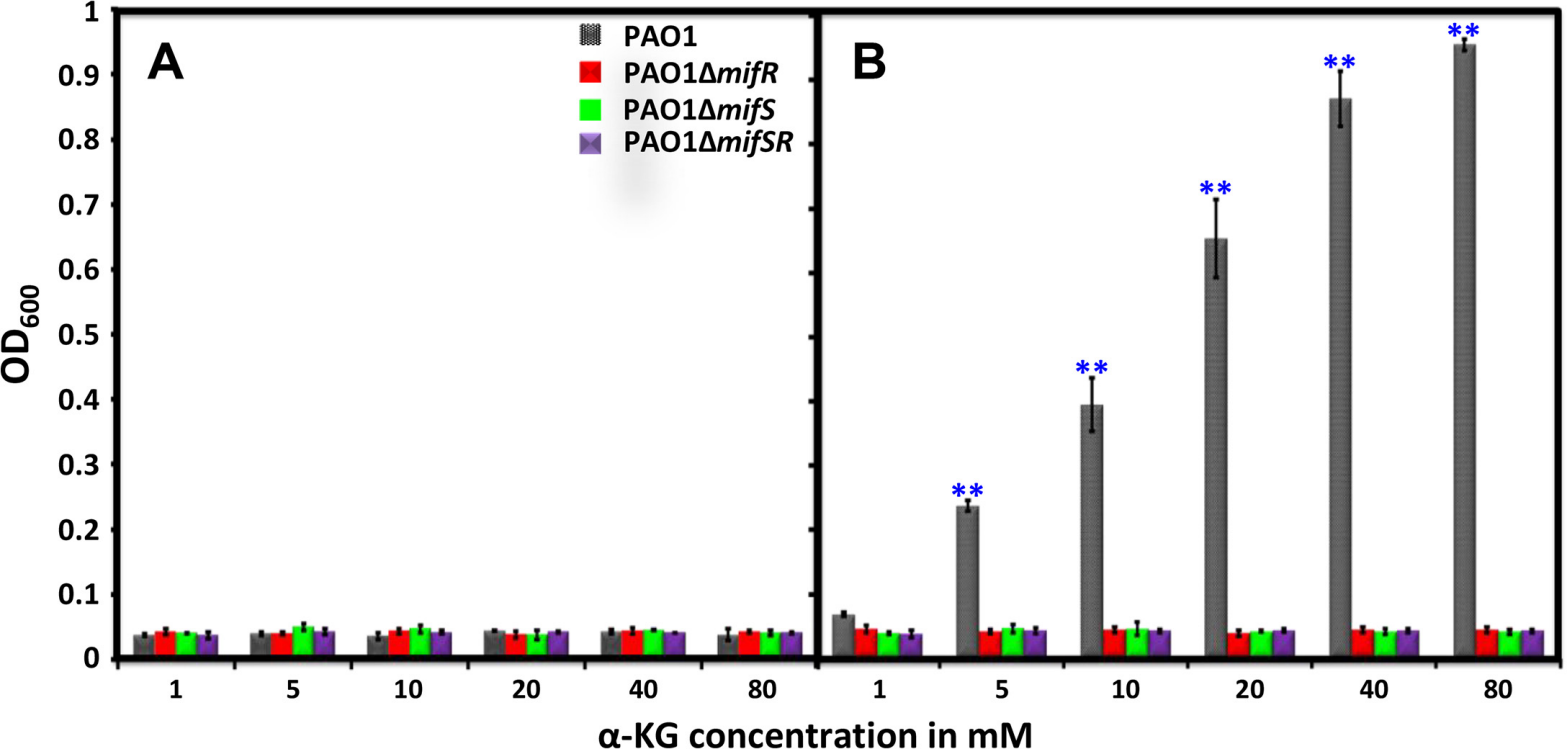
Growth profile in the presence of varying concentrations of α-KG. PAO1 and its isogenic *mifSR* mutants, PAOΔ*mifS*, PAOΔ*mifR* and PAOΔ*mifSR* were grown in M9 minimal media with varying concentrations of α-KG (1 to 80 mM) as the sole carbon source. Growth was monitored by measuring absorbance at 600 nm (OD_600_) over a period of 24 h at 37°C. OD_600_ at 0 h (A) and 24 h (B) is plotted against α-KG concentration. Results shown are mean with standard deviation of three biological replicates. Statistically significant difference between the wild type and mutants as determined by one-way ANOVA with Bonferroni’s post-hoc test, ** *p*-value < 0.001.

**Fig 7:**
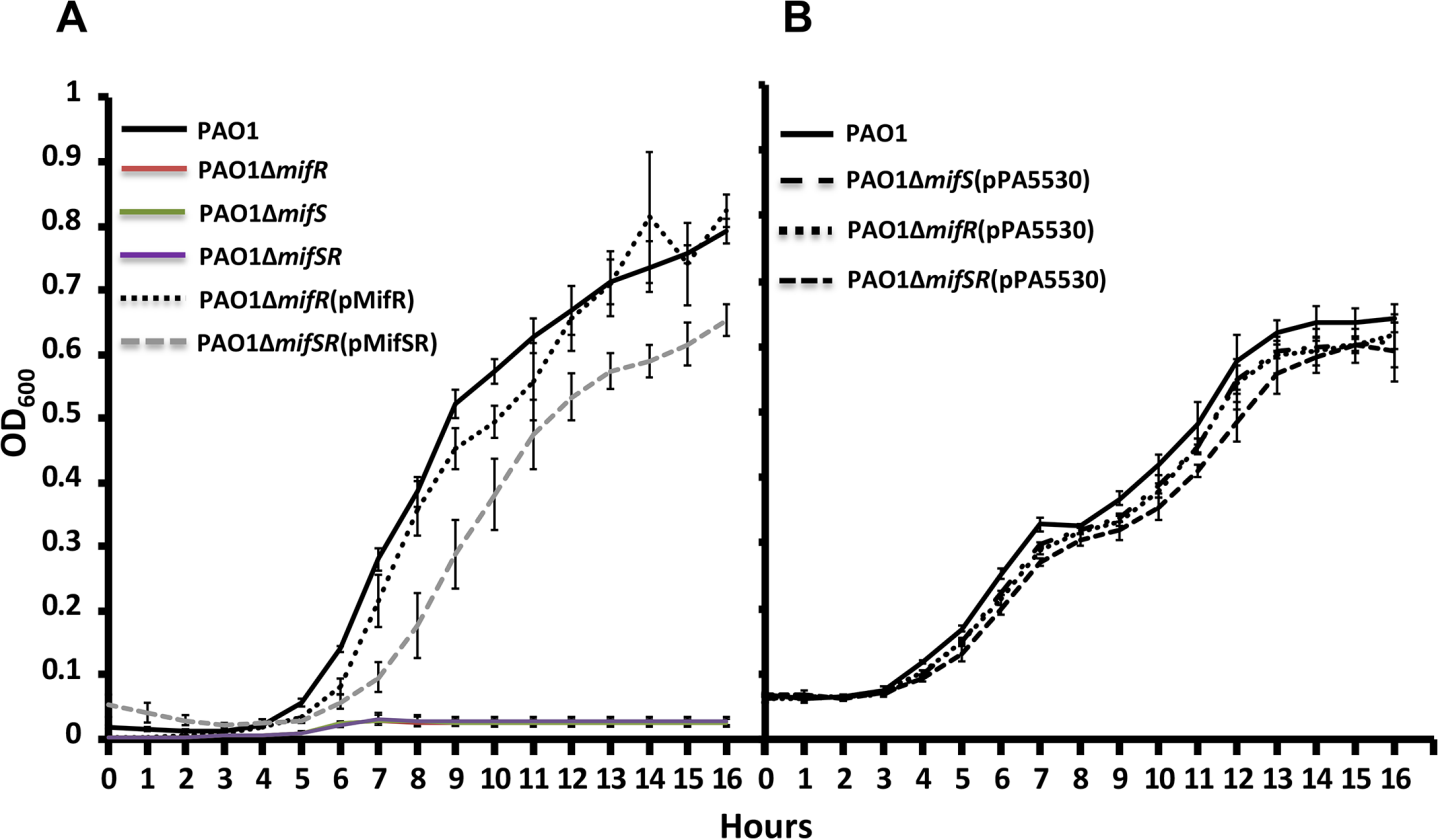
Rescue of α-KG-dependent growth phenotype of *mifSR* mutants. Growth curves of *P*. *aeruginosa* wild-type PAO1, *mifSR* single and double deletion mutants and its complimenting clones (A) and in the presence of pPA5530 (B) in M9 minimal media with α-KG (30 mM).

The inability to utilize α-KG by PAO∆*mifS* (Fig 5A) and PAO∆*mifR* (Fig 5B) in the BioLOG assay was reproduced in M9 minimal media supplemented with 30 mM α-KG (Fig 5C). In fact, all three mutant strains, PAO∆*mifR*, PAO∆*mifS* and PAO∆*mifSR* failed to grow in the presence of α-KG (Fig 5C). To rule out potential toxicity, the wild-type *P*. *aeruginosa* PAO1 and the mutants were cultured in M9 minimal media with varying concentrations of α-KG, ranging from 1 to 80 mM (Fig 6). The mutants exhibited no growth in the presence α-KG after 24 h at 37°C, whereas the wild-type PAO1 exhibited an increase in growth that was proportional to α-KG concentration (Fig 6B). All subsequent experiments were done with 30 mM α-KG. The growth defect exhibited by PAO∆*mifS*, PAO∆*mifR* and PAO∆*mifSR* could be restored to the wild-type levels by introducing *mifR* and *mifSR* genes into the respective mutants (Figs 5D and 7A).

### *mifSR* mutants exhibit α-KG dependent growth defect

**Fig 8:**
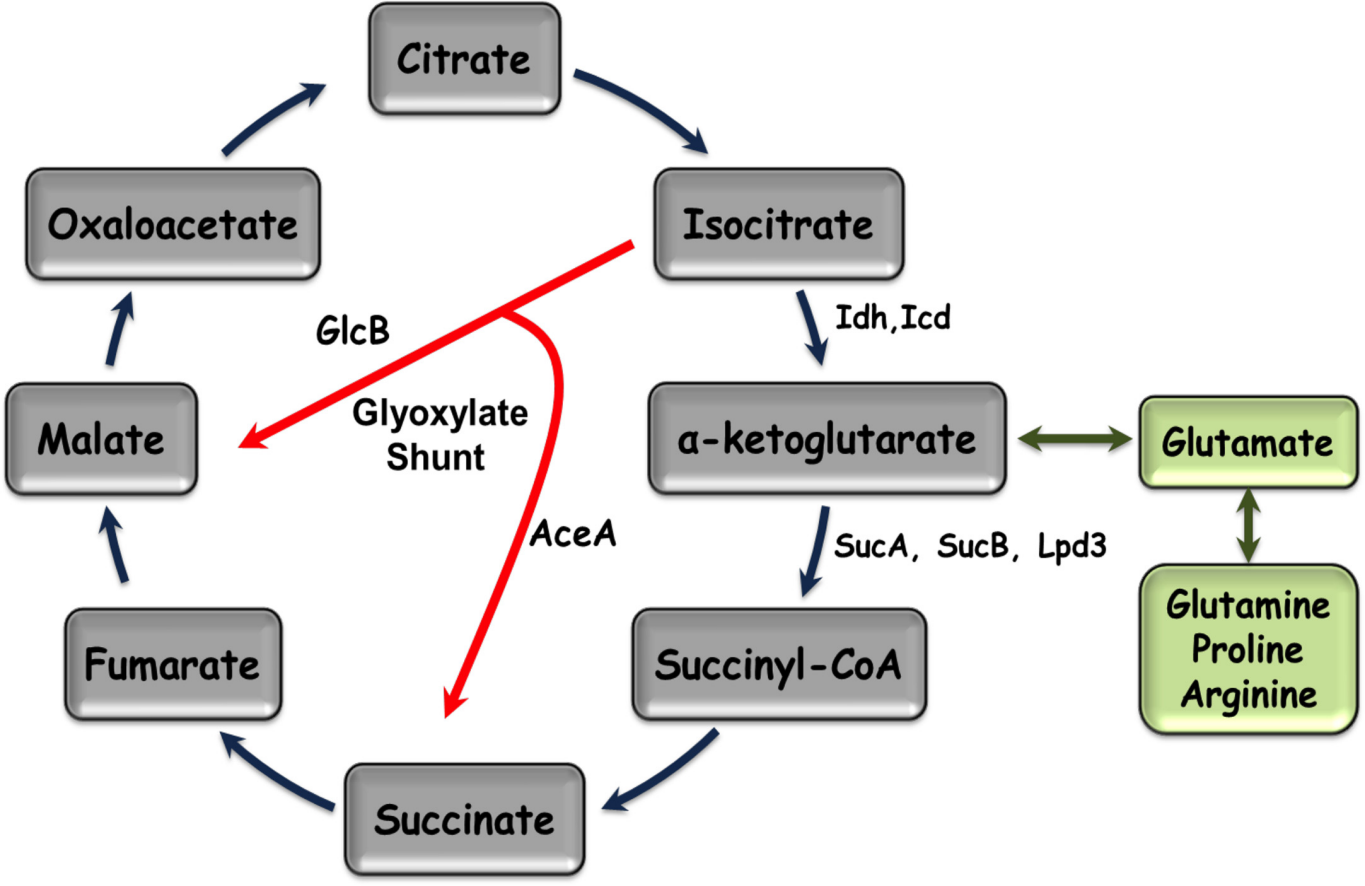
Tricarboxylic acid (TCA) cycle and its related reactions. Enzymes converting iso-citrate to α-KG (iso-citrate dehydrogenase: Icd, Idh), α-KG to succinyl-coA (α-KG dehydrogenase complex: SucA, SucB, Lpd3) and those involved in the glyoxylate shunt (isocitrate lyase (AceA) and malate synthase G (GlnB)) are shown in bold. Green boxes indicate the amino acid biosynthetic precursors of α-KG involved in the anaplerotic reaction.

**Fig 9:**
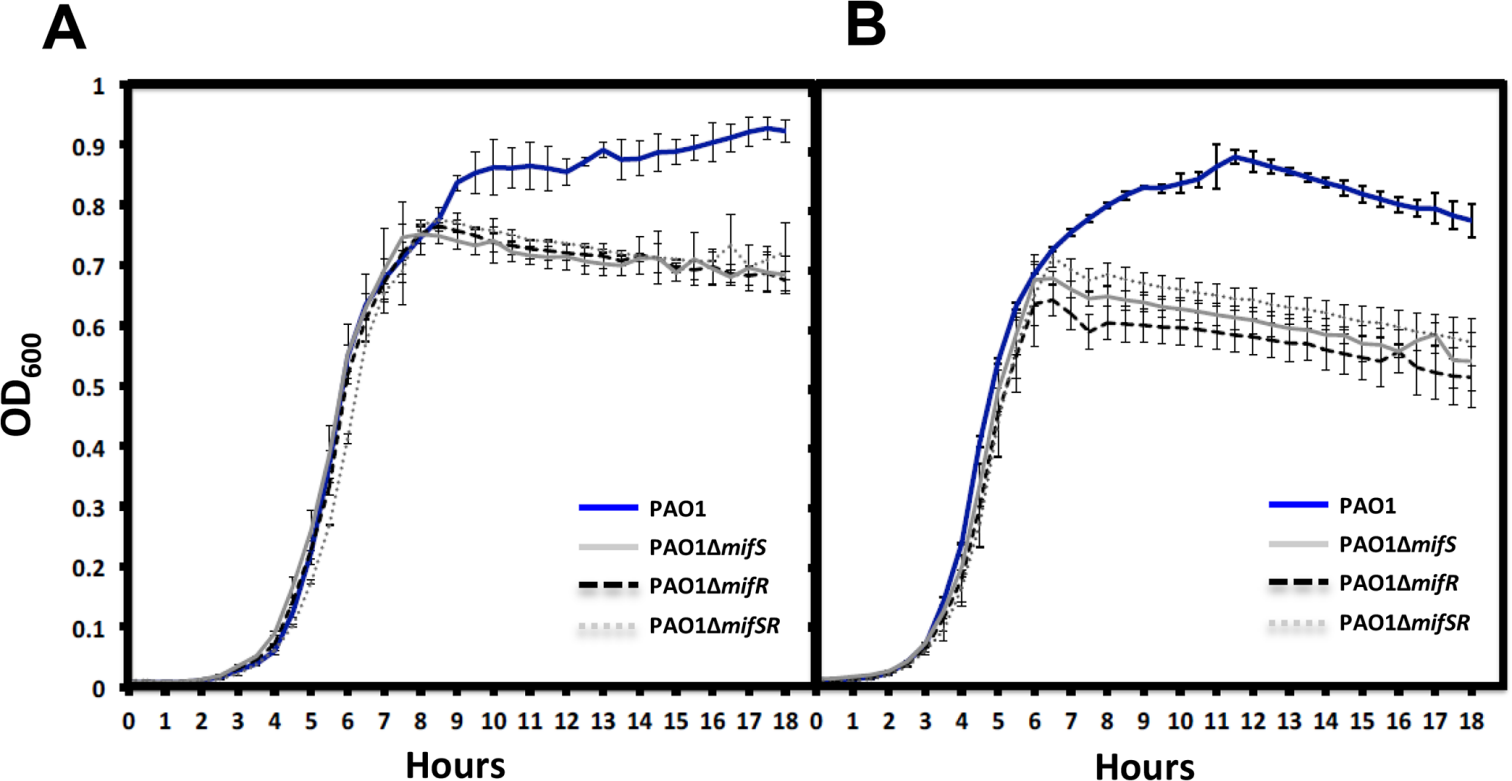
Growth curves in presence of α-KG in combination with succinate and citrate. To determine if α-KG is toxic to the cells, wild-type PAO1 and *mifSR* mutants were grown in the presence of α-KG in combination with succinate (A) and citrate (B) at 30 mM each. In comparison to the wild-type PAO1, *mifSR* mutants shared a similar exponential phase but reached stationary phase earlier, suggesting that it has depleted usable C-source. This suggests that PAO1 can efficiently utilize excess carbon source in the form of α-KG contributing to its increased growth.

α-KG is a key TCA cycle intermediate (Fig 8) and plays an important role in regulating carbon and nitrogen metabolism [44]. It has been previously shown that *P*. *aeruginosa* preferentially utilizes TCA cycle intermediates as a carbon source over other compounds [20,21,45]. To test if the growth defect exhibited by the loss of *mifS* and *mifR* is restricted to α-KG utilization, the mutants and the complementing strains were grown in the presence of TCA cycle intermediates citrate, succinate, fumarate, malate and oxaloacetate at 30 mM each. No difference in growth was observed between wild type PAO1 and its isogenic mutants in the presence of other TCA cycle intermediates except for α-KG (Table 1). This is not surprising as *P*. aeruginosa can use the glyoxylate shunt pathway to bypass the need for α-KG (Fig 8) [46]. Furthermore, no difference in the growth profile of the wild type PAO1 and *mifSR* mutants was observed when grown in the presence of sugars, glucose and sucrose (30 mM each) (Data not shown). To reconfirm that the presence of α-KG is not toxic, the cells were grown in the presence of citrate and succinate combined in equal concentration with α-KG. The mutants and the wild type shared similar early exponential growth (Fig 9). However, the mutants reached stationary phase earlier as compared to the parent strain PAO1. This suggests that the presence of excess carbon source in the form of α-KG further contributes to the growth of PAO1. These analyses indicate that *mifSR* mutants are only defective in α-KG utilization.

**Table 1:** Growth properties of *mifSR* mutants in presence of TCA cycle intermediates.

Carbon Source	PAO1	∆*mifR*	∆*mifS*	∆*mifSR*
**Pyruvate**	**+++**	**+++**	**+++**	**+++**
**Oxaloacetate**	**+++**	**+++**	**+++**	**+++**
**Citrate**	**+++**	**+++**	**+++**	**+++**
**Succinate**	**+++**	**+++**	**+++**	**+++**
**Fumarate**	**+++**	**+++**	**+++**	**+++**
**Malate**	**+++**	**+++**	**+++**	**+++**
**α-Ketoglutarate**	**+++**	**---**	**---**	**---**

+++, growth;---, no growth

Growth of the wild type PAO1 and *mifSR* mutants was tested in M9 minimal media supplemented with different TCA cycle intermediates at 30 mM each, as the sole carbon source. Cells were cultured for 18 to 24 h at 37°C and their growth was monitored by measuring the absorbance at 600 nm. No difference was observed in the growth rate of *mifSR* mutants compared to the parent PAO1 strain. Data is represented in terms of growth and no growth phenotype.

### *mifSR* mutants are defective in α-KG transport

**Fig 10:**
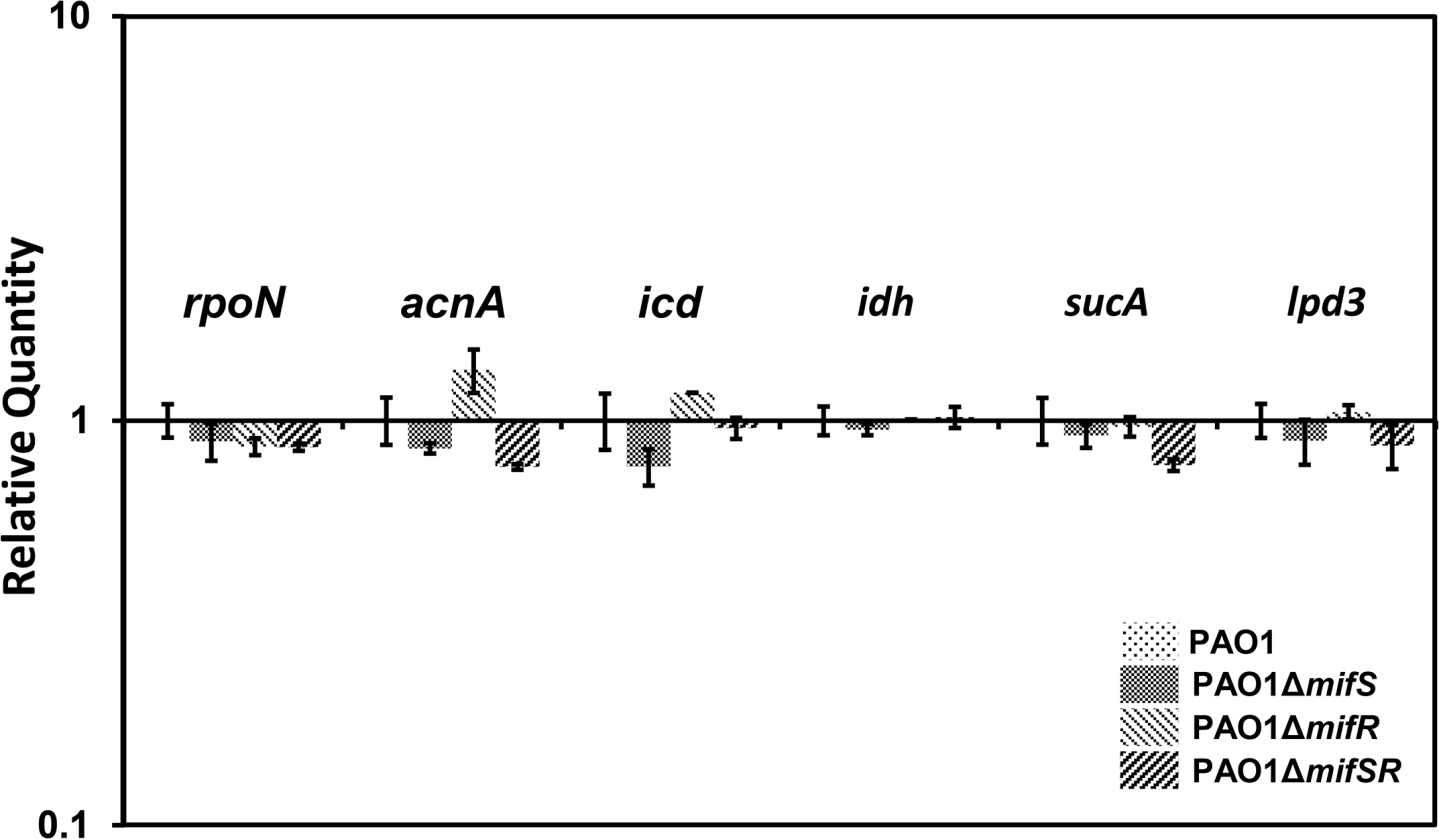
Quantification of *rpoN*, *acnA*, *idh*, *icd*, *sucA*, and *Ipd3* mRNA by qRT-PCR. RNA was isolated from cells grown in M9 minimal media supplemented with citrate (30 mM), reverse transcribed to cDNA and the presence of specific transcripts was analyzed by qPCR using gene-specific primers (Table 5). The expression of genes encoding aconitate hydratase 1 (*acnA* (*PA1562*)) isocitrate dehydrogenase (*idh* (*PA2623*)) isocitrate dehydrogenase, α-KG dehydrogenase complex (*icd* (*PA2623*)), *sucA* (*PA1585)* and *lpd3* (*PA4829*), and σ^54^ (*rpoN* (*PA4462*)) were analyzed in *mifSR* mutants relative to PAO1 (log_10_ RQ = 1). Bars above or below the line represents up- and down-regulation, respectively and the bars are standard errors. The *clpX* (*PA1802*) gene was used as the housekeeping control. Statistically significant difference between the wild type and mutants as determined by one-way ANOVA with Bonferroni’s post-hoc test. Difference in the expression levels of genes is not statistically significant at *p*-value < 0.05.

The absence of growth in the presence of exogenous α-KG could be due to either failure to enter the cells or loss of the mutants’ ability to convert α-KG to succinate. The latter is likely if the mutants failed to express a functional α-KG dehydrogenase complex. The ability of *mifSR* mutants to grow effectively in the presence of citrate and succinate suggests that these mutants are likely to harbor a functional α-KG dehydrogenase complex, unless the mutants bypass it using the glyoxylate shunt (Fig 8). The former is likely as qPCR analysis of genes encoding isocitrate dehydrogenase (*idh*, *icd)* and α-KG dehydrogenase complex (*sucA*, *sucB*, *lpd3)* revealed no difference in the expression levels in the wild-type PAO1 and *mifSR* mutants (Fig 10).

α-KG is a hub for anaplerotic reactions, a process for replenishing TCA cycle intermediates. In this process glutamate, glutamine, proline and arginine act as precursor molecules for α-KG synthesis [47]. Growth studies in the presence of these amino acids would serve as another indirect measure to test the functionality of α-KG dehydrogenase complex in *mifSR* mutants. To test this hypothesis, PAO1, PAO∆*mifR*, PAO∆*mifS* and PAO∆*mifSR* mutants were cultured in the presence of glutamate, glutamine, proline and arginine (Table 2). The parent PAO1 and the isogenic mutants exhibited similar growth phenotype. From the expression studies and growth analyses we deduce that the *mifSR* mutants are impaired in α-KG transport.

**Table 2:** Growth profile analysis of the *mifSR* mutants in presence of amino acids.

Carbon Source	PAO1	∆mifR	∆mifS	∆mifSR
**Glutamate**	**+++**	**+++**	**+++**	**+++**
**Glutamine**	**+++**	**+++**	**+++**	**+++**
**Proline**	**+++**	**+++**	**+++**	**+++**
**Arginine**	**+++**	**+++**	**+++**	**+++**

+++, growth;---, no growth

Cells were grown in the M9 minimal media with the indicated amino acids (30 mM each). Data is represented in terms of growth and no growth phenotype.

### *mifSR* TCS genes regulate extracellular α-KG transport

**Fig 11:**
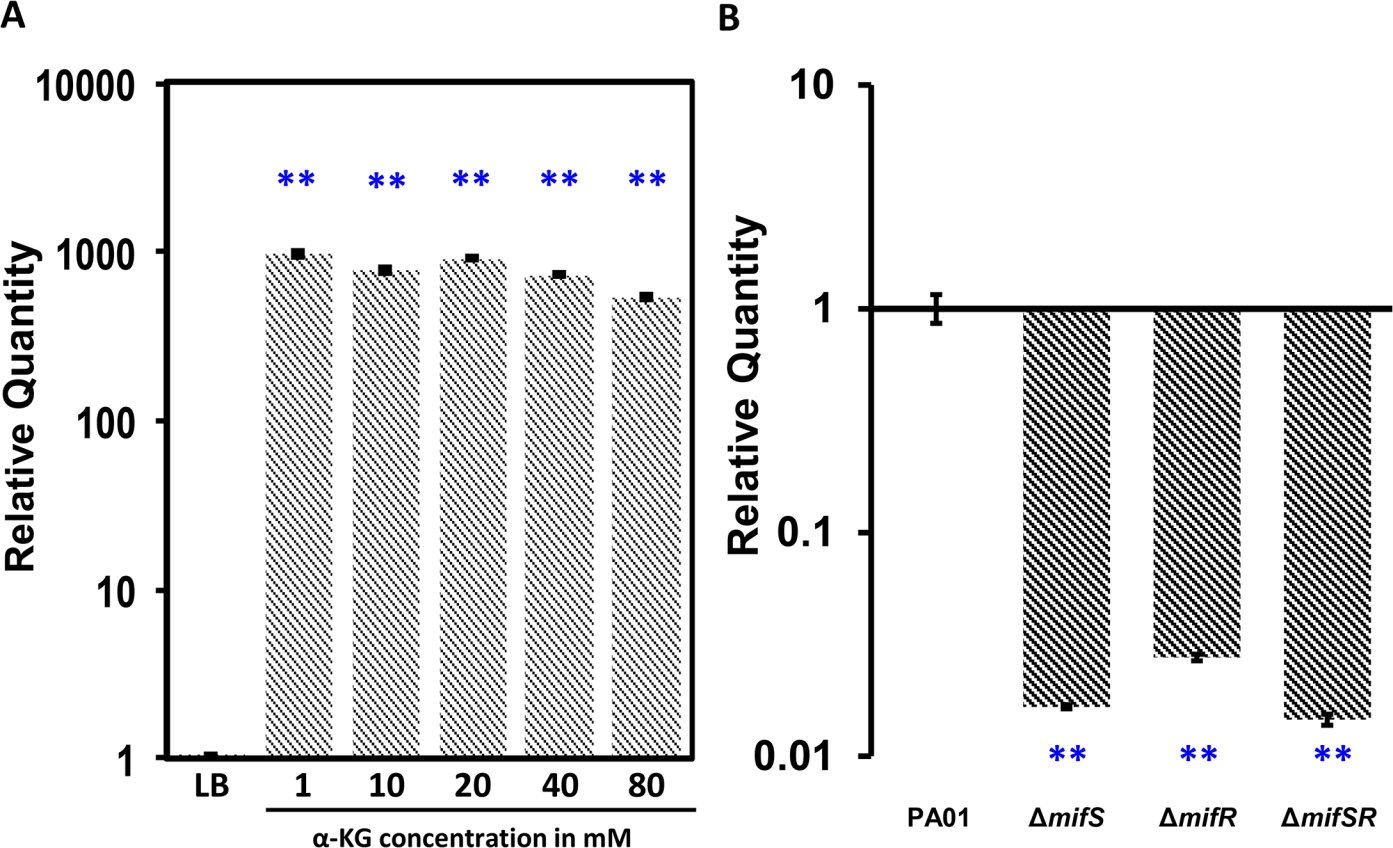
Expression of *PA5530* in response to α-KG. *PA5530* gene expression was determined in the wild type PAO1 with varying concentrations of α-KG (1 h) (A). In addition, the expression of *PA5530* was tested in *mifSR* mutants relative to PAO1, with cells exposed to 30 mM α-KG for 1 h (B). Data was normalized to expression in PAO1 under the respective conditions. Bars above or below the line represents up- and down-regulation, respectively and the bars indicate standard errors. The *clpX* gene (*PA1802*) was used as the housekeeping control. Statistically significant difference between the wild type and mutants as determined by one-way ANOVA with Bonferroni’s post-hoc test, ** *p*-value < 0.001.

In a recent study using transposon mutagenesis; PA5530 was identified as the functional α-KG transporter [48]. To confirm the role of *P*. *aeruginosa PA5530* in α-KG uptake and identify the role of *mifSR* genes, the gene was amplified and subcloned downstream of the inducible P_*lacUV5*_ promoter. The plasmid pPA5530 was introduced into PAO1 and the *mifSR* mutants. Expression of *PA5530* in *trans* in PAO∆*mifS*, PAO∆*mifR*, PAO∆*mifSR* mutants restored their growth to a level similar to the wild-type PAO1 in M9 minimal media with α-KG (30 mM) as the sole carbon source (Fig 7B). Expression of an extra copy of *PA5530* gene in the wild-type PAO1 did not affect its growth (Fig 5E). This finding suggests that expression of *PA5530* is likely regulated by MifSR and/or α-KG. In fact, expression of *PA5530* is regulated by α-KG, as seen in qRT-PCR analysis when PAO1 was grown in M9 media with varying amounts α-KG (Fig 11A). The loss of *mifS*, *mifR* and *mifSR* results in a significant decrease in *PA5530* expression as compared to the wild type PAO1 in the presence of α-KG (Fig 11B). Thus, α-KG-dependent *PA5530* expression requires MifS and MifR.

### RpoN (σ^54^) is required for α-KG utilization

**Fig 12:**
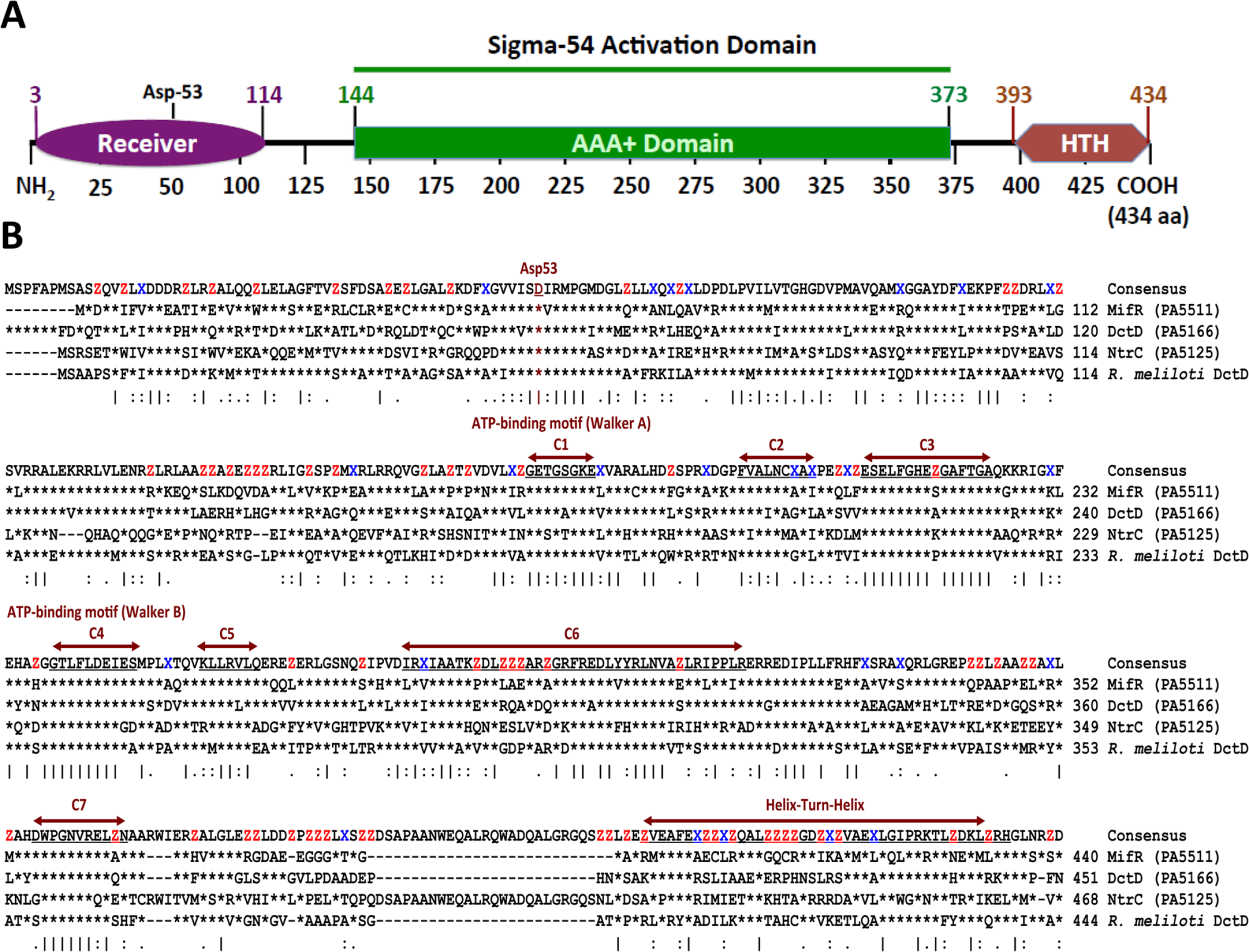
*P*. *aeruginosa* MifR domain organization and sequence alignment. (A) MifR domain organization determined using the Simple Modular Architecture Research Tool (SMART) [51]. MifR is a sigma-54 dependent transcriptional activator [57]. There are three functional domains, N-terminal receiver with the conserved aspartate residue at position 53 (Asp-53) (Purple), central AAA^+^ ATPase, characteristic of sigma-54 dependent activation proteins (Green), and the C-terminal helix-turn-helix (HTH) DNA binding (Red) domains. (B) Sequence alignment of MifR with *P*. *aeruginosa* DctD (PA5166), NtrC (PA5125) and *R*. *meliloti* DctD. Vertical bars indicate conserved residues, asterisk (*) indicate residues are identical at that position. Key residues of the central AAA^+^ domain (C1 to C7) are well conserved amongst sigma-54 dependent transcriptional activators. The horizontal arrow bars indicate HTH domain. Asp-53 indicates the conserved phosphorylation site of *P*. *aeruginosa* MifR. The alignment was generated using ClustalW2 (http://www.ebi.ac.uk/Tools/msa/clustalw2/).

The closest *P*. *aeruginosa* MifS and MifR homologs are *R*. *meliloti* DctB and DctD [40]. In fact, MifR is 69% similar to *R*. *meliloti* DctD that belongs to the Sigma 54 (σ^54^) dependent NtrC family of transcriptional regulators [39,40]. Thus, it is likely that MifR has the conserved domains found among NtrC family of regulators, an N-terminal regulatory, a central σ^54^ activation and a C-terminal DNA binding domains [49,50]. MifR analysis using the simple modular architecture research tool (SMART) [51] and InterPro [52] revealed the presence of three domains: CheY-homologous receiver/regulatory, a central AAA^+^ region required for σ^54^ activation, and the DNA binding helix-turn-helix domains (Fig 12A). The central AAA^+^ domain contains seven conserved regions designated C1 to C7 [50] that are characteristic of σ^54^- dependent transcriptional regulators. Sequence analysis of MifR revealed the presence of all the seven conserved regions in the AAA^+^ domain between amino acid residues 144 to 373 (Fig 12B).

Since MifR exhibits high identity to σ^54^-dependent transcriptional regulators, we hypothesized that *P*. *aeruginosa rpoN* mutants should exhibit a α-KG-dependent phenotype, similar to the *mifSR* mutants. To verify this hypothesis, we tested the ability of PAO∆*rpoN* mutant to grow in the presence of α-KG (30 mM) (Table 3). As expected, PAO∆*rpoN* failed to grow in the presence of α-KG (Table 3). The growth of the *rpoN* mutant was restored in PAO∆*rpoN*::*rpoN* complementing strain. Further, in *trans* expression of *mifR* and *mifSR* in PAO∆*rpoN* mutant failed to restore their growth in the presence of α-KG (Table 3). This data confirms that MifR regulatory function requires functional RpoN (σ^54^).

**Table 3:** Growth properties of PAO1Δ*rpoN* and its derivatives in the presence of α-KG and LB.

Strain	Plasmid	α-KG	LB
**PAO∆rpoN**	**-**	**---**	**+++**
	**Vector**	**---**	**+++**
	**pRpoN**	**+++**	**+++**
	**pMifR**	**---**	**+++**
	**pMifSR**	**---**	**+++**
	**pPA5530**	**+++**	**+++**

+++, growth;---, no growth

Growth of PAO1Δ*rpoN* mutant and its derivatives was tested in the M9 minimal media supplemented with α-KG (30 mM) and in the LB media at 37°C for 24h.

**Fig 13:**
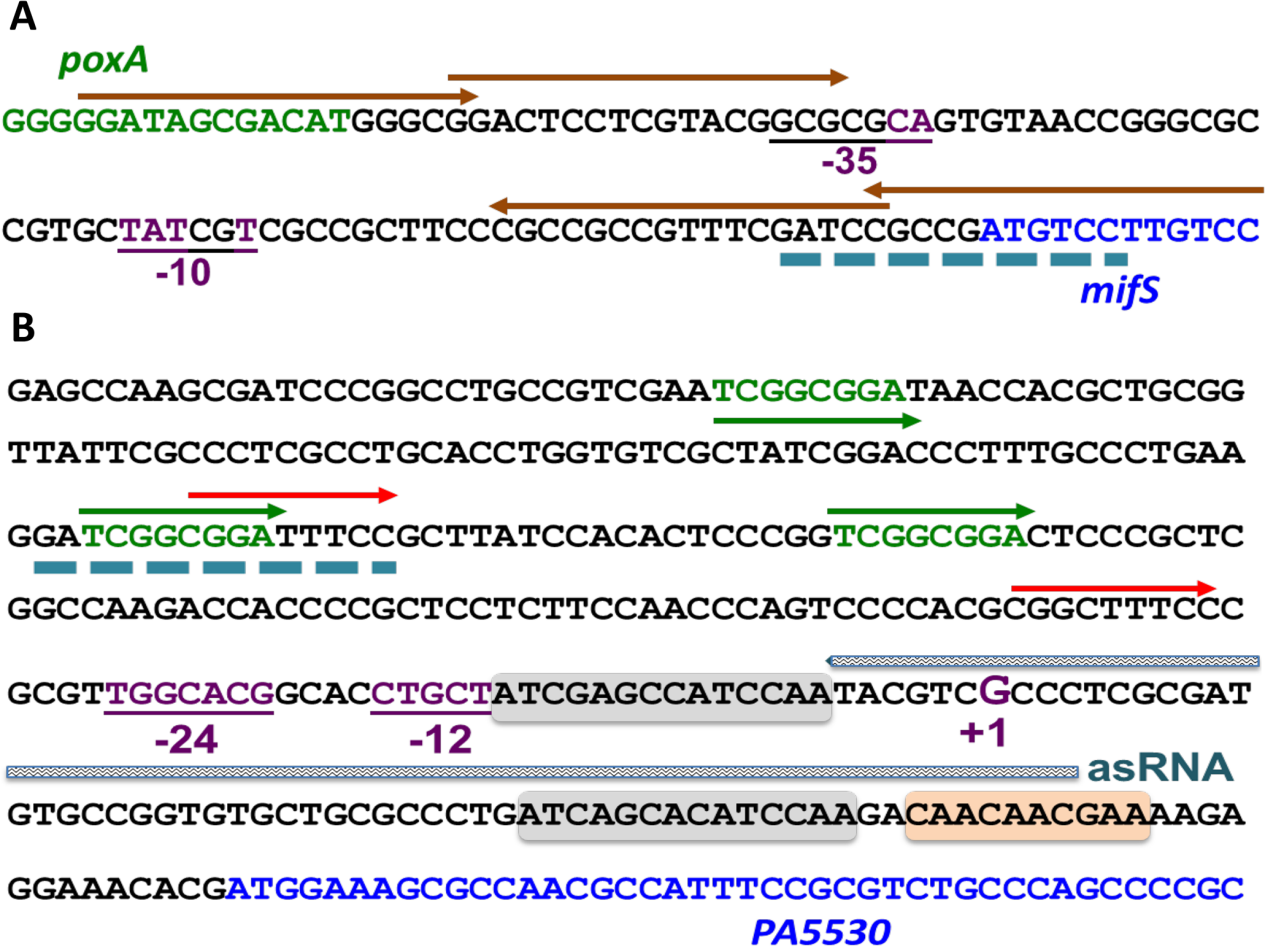
*In silico* analysis of *mifS* (P_*mifS*_) and *PA5530* (P_*PA5530*_) promoter sequences. Motif search was done using the ensemble learning method SCOPE and GLAM2 (Gapped Local Alignment of Motifs) [113,114]. (A) Sequence analysis of the 81-bp (P_*mifS*_) (black) indicates a putative σ^70^-dependent -10 consensus (TATAAT). However, it lacks the -35 consensus (TTGACA) for σ^70^ promoter [80]. Arrows represent the long 17-bp direct and inverted repeats in P_*mifS*_ with a consensus GGAt/cAGCGACATCGGCG. (B) The 315-bp promoter region of *PA5530* showing strong -12 and -24 σ^54^-dependent promoter like element and the proposed transcription start site (+1). Dashed line (blue) depicts a common motif in P_*mifS*_ and P_*PA5530*_ suggesting a common regulatory mechanism (A and B). The three pairs of direct repeats in P_*PA5530*_ are represented by green, blue and orange arrows_._ P_*PA5530*_ possess the signature sequence (AAc/uAAc/uAA) for catabolite repression control (Crc) protein (brown box) [90]. The uncharacterized small antisense RNA (asRNA) identified in the P_*PA5530*_ region [91] is indicated by marked line.

The small 81-bp *mifSR* promoter has no obvious RpoN sigma factor -12/-24 consensus sequence: 5’-TGGCACG-N4-TTGCW-3’ in which W stands for either A or T (Fig 13A) [53]. In fact, it appears to have a potential -10 (consensus: TATAAT) but lacked -35 (consensus: TTGACA) for sigma-70 promoter (Fig 13A) [54]. On the other hand, the promoter region of *PA5530* is 315-bp long with strong -12 and -24 boxes upstream of the predicted transcription start site (Fig 13B). We hypothesized that the inability of *rpoN* mutant to utilize α-KG can be rescued by expressing PA5530 under a regulatable promoter P_*lacUV5*._ As expected, the growth of the *rpoN* mutant was restored when the plasmid harboring the transporter PA5530 was expressed in *trans* (Table 3). This suggests that expression of PA5530 requires both MifSR TCS and RpoN.

The presence of a common motif, GATCGGCGGATt/gTCC, in the P_*mifS*_ and P_*PA5530*_ (Fig 13A and 13B) suggest that these two operons share some common regulatory mechanism. In addition, both promoters possess multiple motifs: P_*mifS*_ has two sets of large overlapping inverted repeats, and P_*PA5530*_ has three sets of direct repeats (Fig 13A and 13B). However, the role of these motifs remains to be elucidated.

## Discussion

*P*. *aeruginosa* pathogenicity relies significantly on its metabolic flexibility. However, establishment of successful infection and its progression requires more than just meeting nutritional demands. Precision in sensing environmental signals concomitant with a quick and appropriate response is the key to efficient bacterial adaptation and survival. An arsenal of TCSs encoded in its genome has furnished *P*. *aeruginosa* with a sophisticated capability to regulate diverse metabolic and virulence processes, ensuring its success as a pathogen [55–57]. *P*. *aeruginosa* genome encodes one of the largest groups of TCS proteins identified in any sequenced bacterial species [57,58]. Bacterial TCS’s sense and respond to a variety of external cues such as nutrient availability, osmolarity, redox state, temperature, and concentrations of other extracellular molecules [59]. However, very few TCS signaling molecules have been identified to date. In this study we suggest that the *P*. *aeruginosa* MifSR TCS exclusively senses α-KG, a C_5_ dicarboxylate and a key component of TCA cycle.

### *P*. *aeruginosa* antibiotic resistance is independent of MifSR TCS

A common feature of bacterial genomes is a close association between the functionally related genes and their location on the chromosome [60,61]. Typically, genes encoding functionally related HKs and RRs are often physically linked and are co-transcribed as an operon [30,62]. Indeed, our *in silico* analysis (Fig 1A and 1B) and cDNA amplification (Fig 1C) reveled that *mifS*-*mifR* genes are co-transcribed and form an operon. This also suggests that HK-MifS and RR-MifR are functionally related and work as a TCS pair. In addition, TCS proteins are known to regulate expression of genes in their immediate vicinity [30]. The *mifSR* genes are 81 bp upstream of the two-gene *poxAB* (*PA5513-5514*) operon. Due to the proximity of *mifSR* to *poxB* which encodes for a β-lactamase, we postulated that *mifSR* TCS regulates antibiotic resistance. However, our initial results nullified this hypothesis in which comparative MIC’s (Data not shown) and qRT-PCR data (Fig 2) showed no difference in antibiotic resistance profiles or *poxB* expression between the wild-type PAO1 and *mifSR* single and double deletion mutants.

### MifSR TCS regulates *P*. *aeruginiosa* α-KG utilization

A previous transcriptome study of the wild-type PAO1 and a *mifR* deletion mutant cultivated under biofilm-specific conditions showed significant alteration in the expression of genes involved in regulating *P*. *aeruginosa* metabolism, small molecule transport and amino acid biosynthesis [42]. The majority of the changes observed in phenotypic microarrays of the *mifS* and *mifR* mutant strains cultivated under planktonic conditions were associated with chemical sensitivity and not with metabolism (Fig 3B). Only 12–16% of phenotypic changes were associated with metabolism. This confirms the significant metabolic differences in the rich planktonic versus anaerobic mode of biofilm growth in *P*. *aeruginosa* [63].

Petrova *et al*. (2012) have also demonstrated that genes involved in energy metabolism, including anaerobic metabolism and fermentative pathways using arginine (*arcDABC*) and pyruvate, were expressed significantly less in *∆mifR* mutant biofilms as compared to its parent PAO1 [42]. Though pyruvate is needed for biofilm formation, it cannot compensate for the loss of *mifR* [42]. Interestingly, the biofilm phenotype associated with the loss of *mifR* can be complemented by *ldhA* encoding D-lactate dehydrogenase to wild type levels of biomass accumulation and microcolony formation [42]. These findings suggest that MifR somehow regulates expression of *ldhA*, a second gene in a three-gene operon *gacS*-*ldhA*-*PA0926* [57]. Importantly, analyses of the promoters reveal the presence of a shared motif in P_*mifS*_ (GATCCGCCGATGTCC) and P_*PA5530*_ (GATCGGCGGATTTCC) (Fig 13) and P_*gacS*_ (AATCCGCCGGGCTGC) suggesting a possible coordinate regulation, and that need to be verified.

Our phenotypic microarray analyses and growth experiments suggested that *P*. *aeruginosa* α-KG utilization requires MifS and MifR (Figs 5 and 7A). The ability of PAOΔ*mifR*, PAOΔ*mifS* and PAOΔ*mifSR* to grow in the presence of α-KG was restored by in *trans* expression of *mifR* and *mifSR* (Fig 7A). Interestingly, the PAO∆*mifS* was complemented by pMifR and pMifSR (Fig 5D) but not by pMifS alone. To rule out that gene expression may have been compromised, the *mifS* gene was cloned downstream of the inducible P_*lacUV5*_ promoter. Though the expression of stable protein was visible in a protein gel, it failed to complement PAO∆*mifS* mutant (data not shown). This suggests that cis-expression of *mifS* and *mifR* is critical for MifS-function. Other researchers have encountered similar problems involving histidine kinases [64]. Moreover, complementation of the PAO∆*mifS* with pMifR suggests that either phosphorylation is not required or there is a potential crosstalk between MifR and other non-cognate HKs. Alternatively, phosphorylation of MifR can occur through small molecule phosphor-donors, like acetyl phosphate, carbamoyl phosphate and phosphoramidate [65]. Such phenomenon is observed with other TCS RRs [66–68]. However, this has to be verified.

The C_5_-dicarboxylate α-KG is an important intermediate in the energy-generating TCA cycle (Fig 8) and plays a key role in regulating carbon and nitrogen metabolism [44]. Similar to other bacteria [69], TCS’s in *P*. *aeruginosa* have been reported to regulate transport and utilization of TCA cycle intermediates such as succinate, fumarate, malate and citrate [39,56]. The *R*. *meliloti* DctB/DctD system is a well-characterized TCS that controls the transport of TCA cycle C_4_-dicarboxylates succinate, fumarate and malate [69]. Though *P*. *aeruginosa* MifS/MifR proteins are homologous to *R*. *meliloti* DctB/DctD TCS proteins, the *mifSR* mutants efficiently utilized citrate, succinate, fumarate, malate, oxaloacetate, sucrose and glucose but exclusively failed to grow in the presence of α-KG (Table 1). This was further supported by another parallel study that shows that α-KG utilization requires MifR [48]. Thus, the *P*. *aeruginosa* MifSR TCS is specifically and uniquely involved in C_5_-dicarboxylate α-KG utilization.

### MifSR TCS modulates *P*. *aeruginosa* α-KG transport

The inability to utilize α-KG suggested that the *mifSR* mutants either have a defective α-KG dehydrogenase complex (inability to convert α-KG to succinyl-coA, Fig 8), or they are deficient in the transport of α-KG into the cell. The former was ruled based upon multiple findings: unchanged expression levels of genes encoding α-KG dehydrogenase, *lpd3* (*PA4829*) and *sucA* (*PA1585*) (Fig 10); the ability to use C_4_ and C_6_ dicarboxylates (Table 1) and C5 family of amino acids such as arginine, proline, glutamine, and histidine (Table 2). The C5 family of amino acids act as biosynthetic precursors of glutamate that ultimately are converted to α-KG by a transamination reaction or through the action of glutamate dehydrogenase [70]. These findings strongly argued that the *mifSR* mutants were defective in their ability to transport α-KG into the cell.

To date, among the identified carboxylate transporters, the C_4_-dicarboxylate transporters have been reasonably well characterized. Based on protein sequence similarity analysis, bacterial C_4_-dicarboxylate transporters are classified into five families, namely, dicarboxylate transport (DctA); dicarboxylate uptake (DcuAB), (DcuC) and (CitT) and the tripartite ATP-independent periplasmic (TRAP) families [69]. Amongst these, DctA transporters, a subgroup of the dicarboxylate/amino acid:cation symporter (DAACS) family [71–73], are extensively studied and are implicated in the transport of C_4_-dicarboxylates in *Echerischia coli* [74], *Bacillus subtilis* [28], *Rhizobium meliloti* [38,75], *Rhizobium leguminosarum* [37,76] and *Corynebacterium glutamicum* [77]. As we were trying to identify the MifSR-dependent transporter Lundgren *et al*., reported that PA5530 is involved in α-KG transport [48]. As predicted, in *trans* expression of *PA5530* was able to restore the ability of *mifR*, *mifS* and *mifSR* mutants to grow in α-KG (Fig 5E). This is further confirmed by the increase in *PA5530* expression in PAO1 in the presence of α-KG (Fig 11A). PA5530 shares no homology with the *P*. *aeruginosa* C_4_-dicarboxylate transporter PA1183 (DctA). However, it does have conserved protein domain family PRK10406 implicated in α-KG transport and shares ~70% homology to *E*. *coli* and *Erwinia* spp. α-KG permease KgtP [78,79]. A common feature in the transport of C_4_-dicarboxylates and other carbon sources in different bacteria is the involvement of TCS mediated regulatory mechanism. Involvement of TCSs, a stimulus-response coupled mechanism, in the transport of C_5_-dicarboxylates suggests a more profound role of α-KG as a signaling molecule.

### *P*. *aeruginosa* α-KG transport requires functional RpoN (σ^54^)

*P*. *aeruginosa* RpoN (σ^54^) is involved in a myriad of functions including expression of virulence factors and nutrient uptake [80]. Functional RpoN is reported to be critical for maintaining a carbon-nitrogen balance in *Pseudomonads* [56,81–84]. Sequence analysis of MifR indicated a requirement of functional RpoN in modulating *P*. *aeruginosa* α-KG utilization. Our study confirms that α-KG utilization in *P*. *aeruginosa* PAO1 requires functional RpoN (Table 3). This phenotype is not strain-specific as phenotypic microarray profiling (BioLOG) of *P*. *aeruginosa* PA14 *rpoN* mutant exhibited a similar phenotype, a significant difference in the ability to utilize α-KG as a carbon source as compared to the wild-type PA14 [85]. An RpoN-dependent phenotype was also observed with citrate and 4-hydroxyphenylacetate utilization [85]. Similarly, utilization of C_4_-dicarboxylates succinate, fumarate and malate in *R*. *meliloti* and *P*. *aeruginosa* also requires the sigma factor RpoN (σ^54^) [37,39,86].

The need for RpoN (σ^54^) to utilize α-KG in *P*. *aeruginosa* can be bypassed by expressing *PA5530* encoding for the transporter under a regulatable promoter but not MifS and MifR. Consistent with the need for RpoN (σ^54^), the promoter for *PA5530* has the requisite signature sequences (Fig 13). Like most complex RpoN-dependent promoters [87], the region is long with multiple motifs that include a signature sequence (AAc/uAAc/uAA) for catabolite repression control (Crc) protein, a post-transcriptional inhibitor that binds the mRNA preventing translation [88–90]. Expression of *crc* is in-turn regulated by RpoN-dependent non-coding RNA CrcZ [90] whose absence in *rpoN* mutant can also lead to reduced expression of *PA5530*. Also, analysis of *P*. *aeruginosa* PA14 transcripts indicates that the *PA5530* promotor is under a small non-coding antisense RNA (asRNA) regulation [91]. Though the role of Crc, CrcZ and the asRNA in α-KG transport has to be verified experimentally, it suggests an additional layer of regulation superimposed on the need for MifS and MifR on the expression of the C_5_-dicarboxylate transporter PA5530.

## Conclusion

**Fig 14:**
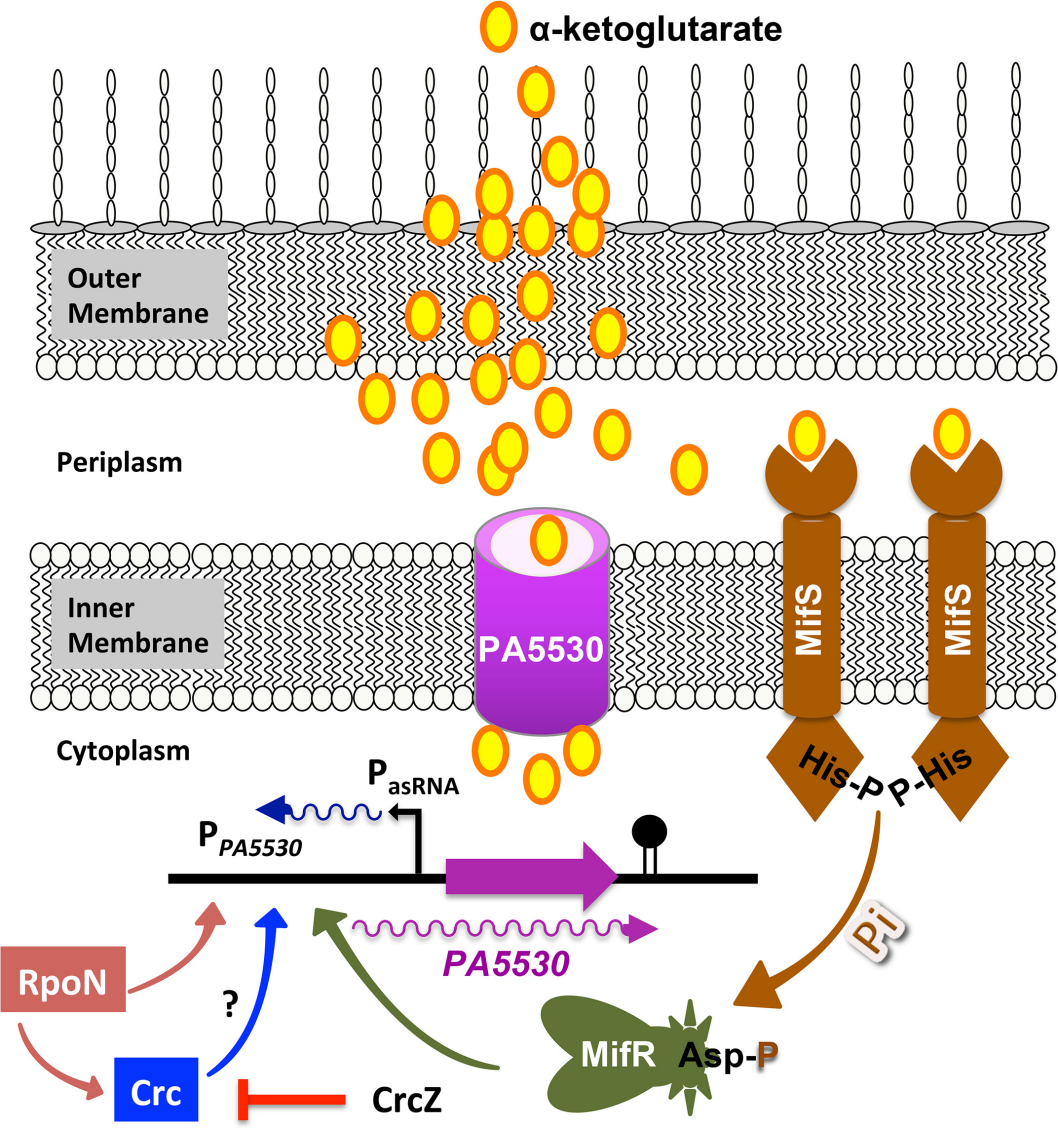
Proposed model for α-KG utilization in *P*. *aeruginosa*. HK-MifS senses the extracellular α-KG to undergo phosphorylation. The phosphate is transferred to the RR-MifR. The phosphorylated MifR in coordination with RpoN (σ^54^) activates the expression of α-KG specific transporter gene *PA5530*. PA5530 thus enables the influx of α-KG to meet the metabolic and energy demands of the cells. *PA5530* promoter (P_*PA5530*_) region has a Crc binding site (Fig 13), suggesting that it is under the catabolite repression control by Crc/CrcZ. The P_*PA5530*_ also shows the presence of another uncharacterized small non-coding asRNA indicating a multilayered and complex regulation of the α-KG transport system.

In eukaryotic cells, the mitochondria serve as a hub and reservoir of the TCA cycle and its intermediates, respectively. Bacterial pathogens can be highly virulent intruders of the host tissue, causing significant damage leading to cellular aberrations and injury. Mitochondrial dysfunction, a consequence of cell injury, results in efflux of TCA cycle intermediates leading to an increase in their extracellular concentrations [92]. It is known that TCA cycle intermediates (C_4_, C_5_, and C_6_ dicarboxylates) are present at micromolar (μM) concentrations in blood that increase with tissues damaged [26,92]. α-KG can also act as a reactive oxygen species scavenger, especially for hydrogen peroxide, protecting both host and pathogen [93]. For pathogenic bacteria such as *P*. *aeruginosa*, efficient uptake of TCA intermediates from the host is crucial for its survival, especially when it is bombarded with host reactive oxygen species, and requires the activity of bacterial carboxylate transport proteins. The transport proteins could be specific for C_4_, C_5_, and C_6_ intermediates and may use a cognate TCS. This study suggests a complex regulatory cascade in modulating *P*. *aeruginosa* C_5_-dicarboxylate, α-KG uptake involving the PA5530 transporter, the MifS/MifR TCS and the sigma factor RpoN (Fig 14). It appears that MifS senses the presence of α-KG and signals MifR. The activated MifR in concert with RpoN initiates the transcription of α-KG-specific transporter gene *PA5530*. Analyses of the published data suggests that the *PA5530* promoter is under several layers of regulation including catabolite repression mediated by Crc/CrcZ [90] and the small non-coding asRNA [91]. Though the asRNA has been identified [91], it has not been characterized. It is not surprising that the *PA5530* expression is potentially regulated by Crc, as it would allow control of transporter(s) in response to the presence of carbon sources in the environment.

In addition to MifSR (PA5512/PA5511), PA1336/PA1335 have been identified to be homologous to the *Rhizobium* C_4_-dicarboxylate transport regulatory DctB/DctD TCS [39,40]. However, the role of PA1336/PA1335 remains to be elucidated. The *P*. *aeruginosa* genome also encodes 19 other paralogs of PA5530 dicarboyxlate transporters, most of which have share less than 50% similarity except for PA0229 (PcaT). PA0229 and PA5530 have 73% similarity. Future studies will determine if the transporters are preferentially or hierarchically upregulated depending on the carbon source. It is also important to note that much of bacterial physiology, particularly of pathogens such as *P*. *aeruginosa* remains a mystery. Metabolic versatility, expression of virulence factors and antibiotic resistance together makes *P*. *aeruginosa* an portentous pathogen. Thus, understanding the physiological cues and regulation would provide a better stratagem to fight the often indomitable infections.

## Materials and Methods

### Strains, media and growth conditions

*P*. *aeruginosa* wild-type PAO1 [40] and its derivatives PAO∆*mifS*, PAO∆*mifR*, PAO∆*mifSR* and PAO∆*rpoN* or *Escherichia coli* strain DH5α were used in this study (Table 4). *Saccharomyces cerevisiae* strain InvSC1 (Invitrogen, Life Technologies, Carlsbard, CA, USA) was used for *in vivo* homologous recombination [94]. Briefly, all bacterial cultures were grown in Luria Bertani (LB) broth (5 g tryptone, 10 g sodium chloride, and 5 g yeast extract per liter) or agar (LB broth with 1.5% agar) (Difco, NJ, USA) or M9 minimal Media (64 g Na_2_HPO_4_-7H_2_O, 15 g KH_2_PO_4,_ 2.5 g NaCl, 5.0 g NH_4_Cl, 20 mM MgSO_4,_ 1 mM CaCl_2_ per liter) [95] at 37°C, unless specified otherwise. Yeast extract-peptone-dextrose media (YEPD: 20 g Bacto Peptone, 10 g yeast extract, 20 g dextrose per liter) was routinely used to culture *S*. *cerevisiae* and synthetic define agar-uracil media was used as selection media for pMQ30 yeast transformants [96]. *P*. *aeruginosa* competent cells were prepared as previously described [97]. For growth curve and complementation studies M9 minimal media supplemented with glucose, sucrose or TCA cycle intermediates including citrate, α-KG, succinate, fumarate, malate or oxaloacetate were used as a sole carbon source at 30 mM each unless specified otherwise. Motility assays were performed in LB media (Difco, NJ, USA). For pyocyanin and proverdine production strains were cultivated in King’s A medium (Difco, NJ, USA) and King’s B medium [98]. Cation-adjusted Mueller Hinton broth and agar (Difco, NJ, USA) was used in MIC assays. For plasmid maintenance, antibiotics were added to growth media when appropriate, at the specified concentrations: *E*. *coli*: ampicillin (Ap) 100 μg/ml, gentamycin (Gm) 15 μg/ml, kanamycin (Km) 20 μg/ml, *P*. *aeruginosa*: Gm 75 μg/ml.

**Table 4:** Strains and plasmids used in this study.

Strain ID	Strain/Plasmid Background	Relevant characteristics	Source
***Escherichia coli***			
DH5α	*E*. *coli*	F^-^ Φ80*lac*ZΔM15Δ (*lacZYA-argF*)U169 *deoR recA1 endA1 hsdR17* (rk^-^ mk^+^) *phoA supE44* λ^-^ *thi-1 gyrA96 relA1*	New England Biolabs
***Saccharomyces cerevisiae***			
INVSc1	*S*. *cerevisiae*	MATa *his3D1 leu2 trp1-289 ura3-52*	Invitrogen
***Pseudomonas aeruginosa***			
PAO1		Prototypic wild type	[40]
PKM900	PAO1	∆*mifS* (*PA5512*)	PAO∆*mifS*; This study
PKM901	PAO1	∆*mifR* (*PA5511*)	PAO∆*mifR*; This study
PKM902	PAO1	∆*mifSR* (*PA5511-PA5512*)	PAO∆*mifSR*;This study
PAOΔ*rpoN*	PAO1	∆*rpoN* (*PA4462*)	[99]
PAO1Δ*rpoN*::*rpoN*	PAO1	∆*rpoN att* Tn*7*::*rpoN*_*aacC1*	[99]
**Plasmids**			
pCR2.1 TOPO		Ap^R^, Km^R^; *colE1* f1 *ori lacZα*	Invitrogen
pRK600		Cm^R^; *colE1 tra* ^*+*^RK2 *mob* ^*+*^	[100]
pRK2013		Km^R^; *colE1 tra* ^*+*^ RK2 *mob* ^*+*^	[101]
pEXG2		Gm^R^; *colE1*, *oriT* mob^+^ *sacB* ^+^	[102]
pMQ30		Gm^R^; *colE1*, *oriT*	[96]
pPSV37		Gm^R^; *colE1 oriT lacI* ^q^ P_*lac*UV5_	[103]
pGDT001	pCR2.1 TOPO	Ap^R^; A ~1.7-kb *Nhe*I-*Xba*I fragment containing *mifS* ORF (*PA5512*) amplified from PAO1 genome using HK_*mifS*F1 and HK_*mifS*R1 primers and cloned into pCR 2.1 TOPO	This study
pGDT002	pCR2.1 TOPO	Ap^R^; A ~1.3-kb *Nhe*I-*Sac*I fragment containing *mifR* (*PA5511*) ORF amplified from PAO1 genome using GDT_*mifR*F1 and GDT_*mifR*R1 primers and cloned into pCR 2.1 TOPO	This study
pGDT003	pPSV37	Gm^R^; The *mifS* ORF subcloned from pGDT001 as an *Nhe*I-*Xba*I fragment into pPSV37	pMifS: This study
pGDT004	pPSV37	Gm^R^; The *mifR* ORF subcloned from pGDT002 as an *Nhe*I-*Sac*I fragment into pPSV37	pMifR: This study
pGDT005	pPSV37	Gm^R^; A ~3.0-kb *Nhe*I-*SacI* fragment containing *mifSR* (*PA5511-PA5512*) ORFs amplified from PAO1 genome using HK_*mifS*F1 and GDT_*mifR*R1 primers and cloned directly into *Nhe*I-*Sac*I-cut in pPSV37	pMifSR: This study
pGDT006	pPSV37	Gm^R^; A ~1.3-kb *Nhe*I-*Sac*I fragmentcontaining *PA5530* ORF amplified from PAO1 genome using GDT_*PA5530F*1 and GDT_*PA5530*R1 primers and cloned directly into *Nhe*I-*Sac*I*-*cut in pPSV37	pPA5530: This study

### Genetic manipulations

Genetic manipulations were carried out using standard techniques [95]. Primers were synthesized by Integrated DNA Technologies, Inc. (Coralville, IA, USA) and are listed in Table 5. Plasmid DNA isolation was carried out using PureLink Hipure Plasmid Miniprep Kit (Invitrogen, Life Technologies, Carlsbard, CA, USA) and agarose gel fragments were purified using Wizard SV Gel and PCR Clean-Up System (Promega, Madison, WI, USA). RNA and cDNA was made using RNeasy Mini Kit (Qiagen Inc. Venio, Limburg, Netherlands) and SuperScript III First-Strand Synthesis System (Invitrogen, Life Technologies, Carlsbard, CA, USA). Restriction endonucleases were from New England Biolabs (Ipswich, MA, USA) and DNA sequencing was carried out at Florida International University (FIU) DNA core and at GENEWIZ Inc (South Plainfield, NJ, USA). All other chemicals were purchased from SIGMA-ALDRICH (St. Louis, MO, USA), AMRESCO (Solon, OH, USA) and Fisher Scientific (Waltham, MA, USA).

**Table 5:** Primers used in this study.

Primer Name	Sequence
HK*mifS*UF	5’-GGAATTGTGAGCGGATAACAATTTCACACAGGAAACAGCTTCAGCTCGACTCCGCCGTCG-3’
HK*mifS*UR	5’-GACGAAGATCACCTGGTCGCCTAGTTAGCTAGCATCGGCGGATCGAAACGGC-3’
HK*mifS*DF	5’-GCCGTTTCGATCCGCCGATGCTAGCTAACTAGGCGACCAGGTGATCTTCGTC-3’
HK*mifS*DR	5’-CCAGGCAAATTCTGTTTTATCAGACCGCTTCTGCGTTCTGATACCGCTCTCATGACCGAA-3’
*mifR*UF1	5’-TTTGAATTCGCCTGGTCGAGCAGCGCA-3’
*mifR*UR1	5’-TTTGCTAGCTCGCTCATGTCG-3’
*mifR*DF1	5’-TTTAAGCTTCTCGGCTTCGACGCCCAT
*mifR*DR1	5’-TTTGCTAGCTCGCGAGGCGTC-3’
*mifSR*UF1	5’- GGAATTGTGAGCGGATAACAATTTCACACAGGAAACAGCTGCGAGCACCAGCGCGCCACT-3’
*mifSR*UR1	5’-TCTCTGACGCCTCGCGAGGGCTGCTCTAGTTAGCTAGCATCGGCGGATCGAAACGGCGGC-3’
*mifSR*DF1	5’-GCCGTTTCGATCCGCCGATGCTAGCTAACTAGAGCAGCCCTCGCGAGGCGTCAGAGA-3’
*mifSR*DR1	5’-CCAGGCAAATTCTGTTTTATCAGACCGCTTCTGCGTTCTGATTACGTGTTCAGCGCGCTG-3’
HK_*mifS*F1	5’-**G** **C** **T** **A** **G** **C**AGA**AGGAGA**TATACCATGTCCTTGTCCCGTCCGCTG-3’
HK_*mifS*R1	5’-TTT**T** **C** **T** **A** **G** **A**TCATGTCGTTACGCTCGTGTC-3’
GDT_*mifR*F1	5’-**G** **C** **T** **A** **G** **C**AGA**AGGAGA**TATACCATGAGCGACCAGGTGATCTTCGTCGAC-3’
GDT_*mifR*R1	5’-TTT**G** **A** **G** **C** **T** **C**TGCTTCAGGCCGGCTCTTCGC-3’
GDT_*PA5530*F1	5’-**G** **C** **T** **A** **G** **C**AGA**AGGAGA**TATACCATGGAAAGCGCCAAC-3’
GDT_*PA5530*R1	5’-TTT**G** **A** **G** **C** **T** **C**TCAATCGGTCGTGATCTTCGAGTGC-3’
GDT_cotransF1	5’-GGTGTTCAGCCTGATCCTGCCGG-3’
GDT_cotransR1	5’-CCGCTTCGCGGATCGTCGCTTC-3’
GDT_cotransF2	5’-GGATCGTCCACGAACTCGGCGGC-3’
GDT_cotransR2	5’-CAGGCGCACCTCGAAGCCGGAC-3’
GDT_p37_SeqF	5’-GACCCGTTTAGAGGCCCCAA-3’
GDT_p37_SeqR	5’-CGTGCTTTACACTTTATGCTTCCGG-3’
*mifR*_seqF	5’-TGGTGCTGGAGAACCGGC-3’
*mifR*_seqR	5’-GCAGTTCAGCGCCACGAAC-3’
*mifS_*SeqF	5’-ATCTGGAACGGCAGTGGAACC-3’
*mifS_*SeqF2	5’-ATCGACGGCGAGTTGCAGCA-3’
*PA5530*_seqF	5’-TCGCGGCATGGAAGAGAC-3’
*PA5530*_seqR	5’-CATGCCGCGACGCAG -3’
DBS_qRT_clpXF	5'- TGCGATTACGATGTGGAGA -3'
DBS_qRT_clpXR	5'- CCCTCGATGAGCTTCAGCA -3'
GDT_qRT_PA*5530*F	5'-CGCAACGCATCAAGTCGAT-3'
GDT_qRT_PA5530R	5'-AGTCGTACCACTCGACCAGGTT-3'
qRT_*rpoN*F	5'-AAATGCGAAAAAGCCATTGAG-3'
qRT_*rpoN*R	5'-CCCTGTGCCTCCAGTAAACC-3'
qRT_*icd*F	5'-GCGACCGGTGACAAAATCAC-3'
qRT_*icd*R	5'-GGGTTCTTCGGTACGCTCAA-3'
qRT_*idh*F	5'-GGCGATGATCCGCAACTC-3'
qRT_*idhR*	5'-GCATTACCGCCTTGGTGTCT-3'
qRT_s*ucA*F	5'-CTGCAGCCAGCATCACATG-3'
qRT_*sucAR*	5'-CGAGATTGAGGCCCTTCTTG-3'
qRT_*lpd3*F	5'-CATGCGGCGGAGATGAAC-3'
qRT_*lpd3R*	5'-ACTTCCGGCTGGGTGTAGATG-3'

qRT in the primer name indicates that the primer was designed for qPCR. Broken and continuous lines below the primer sequence indicate ribosome binding and restriction sites respectively.

### Construction of *P*. *aeruginosa* ∆*mifR* mutant

An unmarked *mifR* clean in-frame deletion mutant of *P*. *aeruginosa* was generated by gene splicing [104]. Upstream and downstream flanking regions of *mifR* were amplified by PCR (GC Rich PCR System, Roche, Indianapolis, IN, USA), using primers listed in Table 5. A 754-bp P1 and a 720-bp P2 were amplified using upstream primers *mifR*UF1-*Eco*RI and *mifR*UR1-*Nhe*I and the downstream primers *mifR*DF1-*Nhe*I and *mifR*DR1-*Hin*dIII (Table 5), respectively from PAO1 genomic DNA. After sequencing to ensure fidelity, P1 and P2 were spliced together to obtain a 1474-bp deletion fragment with a deletion of *mifR* containing stop codons at its junction (inserted as part of *Nhe*I site in the primer). This was then sequenced and subcloned into a *P*. *aeruginosa* non-replicative plasmid pEXG2 [102] as a *Eco*RI-*Hin*dIII fragment and moved into the wild-type PAO1 strain by allelic replacement [105] using pRK600 and pRK2013 as the helper plasmids [100,101]. Clones were screened for Gm sensitivity (75 μg ml^−1^) and sucrose resistance (8% sucrose) corresponding to a double cross-over recombination event and replacement of the target gene with the deletion product. The presence of the deletion in PAOΔ*mifR* (PKM901) was confirmed by PCR amplification and sequencing of the deletion product (data not shown).

### Construction of *P*. *aeruginosa mifS* and *mifSR* mutants

The unmarked *mifS and mifSR* deletion in PAO1 was generated by using the yeast system of double-stranded gap repair and homologous recombination [106]. Briefly, the *mifS* and *mifSR* upstream and downstream flanking regions were amplified by PCR using primers listed in Table 5. To create a *mifSR* deletion, an upstream 933-bp P1 and a downstream 1115-bp P2 were amplified using primer pairs *mifSR*UF1-*mifSR*DF1 and *mifSR*UR1*-mifSR*DR1, respectively. Similarly, to create *mifS* deletion, an upstream 703-bp P1 and a downstream 653-bp were amplified using primer pairs HK*mifS*UF-HK*mifS*DF and HK*mifS*UR-HK*mifS*DR, respectively. HK*mifS*UF and *mifSR*UF1 primers had stretches of homologous DNA, 5’-GGAATTGTGAGCGGATAACAATTTCACACAGGAAACAGCT-3’ and 5’-CCAGGCAAATTCTGTTTTATCAGACCGCTTCTGCGTTCTGAT-3’, respectively, to target recombination of the amplicons with pMQ30 vector. These primer pairs also had complementing sequences at the 3’ end to facilitate joining to create the P3 fragment, as well as stop codons (CTAGTTAGCTAG) to prevent any run off translation. The pMQ30 vector has double selection markers *URA3* for yeast and gentamycin for *E*. *coli* [96]. Yeast cells were transformed with the P1, P2 and linearized pMQ30 (*Bam*HI digested) using standard protocols [106] and colonies were selected on sucrose-uracil plates.

The yeast colonies were checked for the presence of P3 constructs for *mifS* and *mifSR* deletions by amplification using upstream forward (*mifSR*UF1 and HK*mifS*UF, respectively) and downstream reverse (*mifSR*DR1 and HK*mifS*DR, respectively) primers. Yeast DNA was isolated from the positive colonies as described earlier [106]. *E*. *coli* was transformed with the recombinant pMQ30 plasmids containing P3s and screened for gentamycin resistance. The amplified P3s from the recombinant plasmids were sequenced to ensure fidelity. The constructs were then moved into PAO1 strain using tri-parental mating and screened for single and double crossovers using counter selection with sucrose and gentamycin as described earlier [107,108]. The presence of the gene deletions in all the mutants were confirmed using standard molecular methods (PCR and DNA sequencing of the locus). These strains are henceforth referred to as PAOΔ*mifS* (PKM900) and PAOΔ*mifSR* (PKM902).

### Construction of complementing plasmids

DNA fragments from *P*. *aeruginosa* PAO1 with *mifS* (~1.77 kb) and *mifR* (~1.35 kb) were PCR amplified using primer pairs HK_*mifS*F1-HK_*mifS*F1R1, GDT_*mifR*F1-GDT_*mifR*R1, respectively. In order to ensure expression of the genes, the primers are designed such that the ORF will juxtapose against a strong ribosome binding site [70]. The PCR amplified products were cloned into pCR2.1 TOPO (Invitrogen, Life Technologies, Carlsbard, CA, USA) using manufacturers protocol to generate plasmids pGDT001 and pGDT002, respectively. The fidelity of the PCR amplified product was confirmed by sequencing. The fragments carrying *mifS* and *mifR* were moved into a broad host range pPSV37-Gm plasmid [103] as a *Nhe*I-*Sac*I fragments, downstream of an inducible P_*lacUV5*_ promoter to generate plasmids pGDT003 and pGDT004, respectively. Henceforth, these plasmids are referred to as pMifS and pMifR.

DNA fragments from PAO1 with *mifSR* (~3.12 kb) and *PA5530* (~1.3 kb) were PCR amplified using primer pairs HK_*mifS*F1-GDT_*mifR*R1 and GDT_*PA5530*F1-GDT_*PA5530*R1 (Table 5), respectively. The PCR amplified products were cloned directly into pPSV37-Gm plasmid as *Nhe*I-*Sac*I fragments, downstream of an inducible P_*lacUV5*_ promoter to generate plasmids pGDT005 and pGDT006, respectively. Sequence fidelity was confirmed by sequencing using the primers GDT_p37_SeqF-R, *mifR*_seqF-R, *mifS*_seqF-F2 and *PA5530*_seqF-R (Table 5). Henceforth, these plasmids are referred to as pMifSR and pPA5530.

These expression plasmids were then introduced into wild-type PAO1, PAO∆*mifS*, PAO∆*mifR*, PAO∆*mifSR* and PAO∆*rpoN* deletion mutants by electroporation [97] and gentamycin resistant colonies were selected.

### Phenotypic microarray

Comparative phenotypic microarray profiles of wild-type PAO1 with PAO∆*mifR* and PAO∆*mifS* mutant were performed at BioLOG Inc. (Hayward, CA, USA). Phenotypic profiling was carried out in triplicate and data analyses was done using OmniLog PM software.

### Growth curves

*P*. *aeruginosa* PAO1 and its derivatives were grown overnight at 37°C in LB broth with or without antibiotics. Overnight cultures were washed with sterile 0.85% NaCl (wt/vol) solution to remove spent and residual media. Cultures were diluted in fresh M9 minimal media to obtain equal optical densities (OD_600_) of 0.025. Growth of the cultures was assessed in LB broth and in M9 minimal media supplemented with glucose (30 mM), sucrose (30 mM) or TCA cycle intermediates including citrate, α-KG, succinate, fumarate, malate or oxaloacetate (at 30 mM, unless specified otherwise) as a sole carbon source in 48 and 96 well plates (Falcon). Growth was monitored by determining absorbance at 600 nm using BioTek Synergy HT (Winooski, VT, USA) plate reader for 18–24 h at 37°C. All experiments were performed multiple times in triplicate.

### Pyocyanin and pyoverdine production

Extracellular pyocyanin was quantified by extracting the pigment from culture supernatants using the chloroform-HCL method as described previously [109]. Briefly, 5 ml culture supernatants from stationary-phase cultures (∼18 h) grown in King’s A medium was extracted with 3 ml chloroform. Pyocyanin was then re-extracted into 1 ml of 0.2 N HCl, resulting in a pink color, indicating the presence of pyocyanin that was read at 520 nm. The concentration is expressed as μg of pyocyanin produced per ml of culture (μg/ml), by multiplying the optical density OD_520_ by 17.072 [109].

To measure pyoverdine production, cells were grown overnight at 37°C in King’s B medium [98]. Pyoverdine in the supernatant was read at 405 nm and normalized to the initial cell density (OD_600_). Pyoverdine levels were expressed as a ratio of OD_405_/OD_600_ [110].

### Minimum Inhibitory Concentration

MICs were determined using the E-test as per the manufacturers protocol (BioMerieux, USA) and/or by standard broth microdilution method [111]. The assays were performed in triplicate, each with technical triplicate, for each antibiotic in cation-adjusted Mueller Hinton broth.

### RNA isolation, cDNA synthesis and qRT-PCR

RNA was isolated from *P*. *aeruginosa* wild-type PAO1, PAO∆*mifR*, PAO∆*mifS* and PAO∆*mifSR* strains grown in LB broth followed by 1 h treatment with 30 mM α-KG. Briefly, overnight cultures grown in LB broth at 37°C were washed with sterile 0.85% saline solution to remove spent media and were subcultured at 37°C, 200 rpm in LB media. LB broth was used as a carbon source for initial growth of cultures since PAO∆*mifR*, PAO∆*mifS*, PAO∆*mifSR* and PAO∆*rpoN* strains exhibit growth defects in the presence of α-KG alone. When the cells reached an optical density at 600 nm (OD_600_) of 0.6–0.7 all the cultures were treated with 30 mM α-KG for 1 h. Post treatment, RNA was stabilized by addition of phenol-ethanol mixture [112]. Stabilized RNA was then isolated using RNeasy Mini Kit (Qiagen, Inc Venio, Limburg, Netherlands) as per manufacturer’s protocol. Residual genomic DNA contamination was removed using RQ1 Rnase-free DNase (Promega, Madison, WI, USA) and RNA was repurified using Rneasy Mini Kit (Qiagen, Inc Venio, Limburg, Netherlands). Quality of purified RNA was assessed on a denaturing agarose gel (NorthernMax Gly, Ambion, Life Technologies, Carlsbard, CA, USA) and quantified at 260 nm (BioTEK, Synergy HT, Winooski, VT, USA). cDNA was then synthesized by annealing NS5 random primers to total purified RNA and subsequent extension was carried out using SuperScript III reverse transcriptase (Invitrogen, Life Technologies, Carlsbard, CA, USA).

qRT-PCR to study expression levels of *PA5530* under α-KG induction was performed using Applied Biosystems Step One cycler and detection system with PowerSYBR Green PCR MasterMix with ROX (Applied Biosystems, Life Technologies, Carlsbard, CA, USA). In addition RNA was isolated from PAO1, PAO∆*mifR*, PAO∆*mifS* and PAO∆*mifSR* strains grown in M9 Minimal media supplemented with citrate (30 mM) without α-KG treatment, as described previously. qRT-PCR to study expression levels of genes encoding sigma-54 *rpoN* (*PA4462*), iso-citrate dehydrogenase (*idh* (*PA2623*) and *icd* (*PA2624*)), α-KG dehydrogenase complex (*sucA* (*PA1585)* and *lpd3* (*PA4829*)) were done essentially as described above. The cycling conditions used were 95°C/2 minutes (holding); 40 cycles of 95°C/15 sec, 60°C/1 min (cycling); 95°C/15 sec, 60°C/1 min, 95°C/15 sec (0.6°C ramp) (melt curve). Expression was normalized to *clpX* (*PA1802*), whose expression was determined to remain constant between the samples and conditions tested [107].

### Bioinformatic Analyses

Sequence analyses and domain organization studies were performed using the Simple Modular Architecture Research Tool (SMART) [51] and InterPro domain prediction database [52]. *mifS* (P_*mifS*_) and *PA5530* (P_*PA5530*_) promoter analyses and motif search was done using the ensemble learning method SCOPE and GLAM2 (Gapped Local Alignment of Motifs) [113,114]. Multiple sequence alignment was generated using ClustalW2 (http://www.ebi.ac.uk/Tools/msa/clustalw2/) and www.pseudomonas.com [57].

### Statistical Analyses

All data were analyzed for statistical significance using the Student’s *t*-test on GraphPad or Analysis of Variance (ANOVA) with post-hoc testing when appropriate, on IBM SPSS Statistics 22.0 statistical analysis software. Differences were considered to be significant at *p*- values < 0.05.
